# Financing opportunities in the time of COVID-19: Re-examining cigarette taxes with a new scorecard

**DOI:** 10.18332/tid/133441

**Published:** 2021-03-10

**Authors:** Francis Thompson, Frank J. Chaloupka, Susan Sparkes, Jeffrey Drope, Erika Siu, Margaret Dorokhina

**Affiliations:** 1 HealthBridge Foundation of Canada, Ottawa, Canada; 2 Tobacconomics, Health Policy Center, University of Illinois at Chicago, Chicago, United States; 3 Department of Health Systems Governance and Financing, World Health Organization, Geneva, Switzerland

**Keywords:** COVID-19, tobacco, cigarette taxes, tax policies, earmarking

The 18th WCTOH has announced its plan to hold a virtual Leadership Summit on Tobacco Control from 6–7 May 2021. Ahead of the Summit a series of open-access webinars is being held to provide on-going access to expert opinion on issues of importance to the Tobacco Control community. The third of these webinars entitled ‘Fiscal strategies for financing health services in pandemic times: the case for tobacco tax’ was held on 15th December 2020.

During the webinar, S. Sparkes, World Health Organisation, Switzerland, and F. J. Chaloupka, University of Illinois Health Policy Centre, United States, were the invited expert speakers.

With governments worldwide focused on the COVID-19 pandemic and its economic impacts, tobacco control may seem far off the agenda for the next few years. But the economic fallout of the pandemic and the stark realization of the need to invest more in health can be good for one tobacco control intervention: higher and more effective cigarette taxes. Most countries in the world have considerable room to improve their tobacco tax policies.

That is the conclusion of a recent discussion on health financing and cigarette taxes, held in mid December 2020 as part of a webinar series hosted by the 18th World Conference on Tobacco or Health.

S. Sparkes, World Health Organization economist, provided a sobering overview of the dramatic impact the pandemic is having on economies around the world, and in turn on government finances. The pandemic has caused a sudden, sharp and global drop in GDP that makes the financial crisis of 2008 look small in comparison^1^. As GDP has contracted, governments have had to take on additional debt to cope with the crisis. These dynamics, coupled with increasing poverty rates, will have long-term impacts from which it will take years to recover^2^.

At the same time, the pandemic has stretched health systems and highlighted how much investment is still needed to ensure equitable access to quality health services without financial hardship (i.e. Universal Health Coverage). For several years after the acute phase of the pandemic is finished, governments will be facing a simultaneous increase in demand for public spending and reduction in the revenue base to pay for it.

This fiscal squeeze should make Ministries of Finance more open to health taxes that simultaneously generate revenue for the state and directly improve citizens’ health by reducing consumption of unhealthy products. The archetypal and best-researched form of health tax is cigarette excise taxation, as explained by F. J. Chaloupka of the Health Policy Center at University of Illinois at Chicago, USA.

F. J. Chaloupka unveiled a new tool to rate governments on their policies, the Cigarette Tax Scorecard. The global average score is only 2.07 out of 5, suggesting that governments have ample opportunity to improve their tax policies and boost their tobacco tax revenues. The top performing countries, New Zealand and Australia, are faring significantly better and score 4.63, with high scores in each component. Comparing the 2018 scores with those of 2014 shows only a modest increase in the global average, partially due to 43 countries that actually saw a decrease in their performance. On the other hand, the largest improvements were seen in Bahrain, Saudi Arabia, United Arab Emirates, Kyrgyzstan, and the Philippines.

The Scorecard uses data compiled in the World Health Organization's biennial reports on the global tobacco epidemic to assess cigarette tax policies in over 170 countries in 2014, 2016, and 2018. These scores are representative of the current strengths and opportunities for improvement in the tax policies of each country and provide a roadmap for the future, while allowing comparisons across the globe.

Four key dimensions are used to determine how well governments are taxing cigarettes: cigarette price, changes in cigarette affordability over time, share of taxes in retail cigarette price, and cigarette tax structure. Global evidence shows that these components are the best indicators of the effectiveness of cigarette tax policies.

Cigarette prices are a key indicator of tobacco use, as substantial price increases remain the most effective way to reduce consumption^3^. To compare countries with varying income levels, the Cigarette Tax Scorecard uses the purchasing power parity price for the most sold brand. The optimal score of 5 is awarded to countries where the price of a cigarette pack is at least ten international dollars. The global average score for this measure is 2.35, leaving ample room for improvement.

As economies and incomes grow, governments need to update and increase taxes on cigarettes in order to continue to decrease affordability^4^. The Scorecard assesses changes in affordability over the last six years. For this component of the score, the maximum score is awarded to countries that implemented at least one excise tax increase between 2012 and 2018, resulting in a statistically significant annual average change in affordability of 7.5% or higher. Countries around the world are not performing well on this measure, as the global average is only 1.18.

The share of taxes in cigarette prices is essential to ensure that governments continue to increase tax revenue collection from cigarettes, while decreasing consumption^5,6^. In addition to the general tax share in cigarette prices, the share of excise taxes is another important factor, as excise taxes have the biggest impact on consumption^7^. In the Scorecard, this component score is calculated as an average of the total tax share score and the excise tax share score. Countries with a minimum of 70% excise share and a minimum of 75% total tax share receive 5 points. The global average score of 2.06 leaves much room for improvement, although countries in the European region receive a significantly higher score of 3.40.

The final component of the Cigarette Tax Scorecard is tax structure, as this has a significant impact on the variability of prices of cigarettes. The highest score was awarded to systems with either a uniform specific excise tax that is automatically adjusted, or a mixed excise tax with an emphasis on the specific component in addition to a minimum tax, an automatic adjustment to the specific tax component, and the use of the retail price as the base for the ad valorem component^4^. The average score of countries in this metric is 2.69, with substantial variations between regions.

To summarize: there is considerable untapped potential for governments to use health taxes, and especially cigarette taxes, to increase government revenues at a time of fiscal contraction. Reducing tobacco use results in a significantly healthier population and a less burdened health system. Furthermore, the additional revenue that these taxes bring can be used by countries to assist in economic recovery after the pandemic. The new Tobacconomics Cigarette Tax Scorecard provides policy makers and advocates with an objective, actionable assessment of current cigarette tax policies, which can be used to reach a country’s public health and economic goals in the future.
