# Supplementary material for: Financing opportunities in the time of COVID-19: Re-examining cigarette taxes with a new scorecard
Source: Tob Induc Dis. 2021 Mar 10;19:18. doi: 10.18332/tid/133441 (PMC7944517; doi:10.18332/tid/133441)
Supplement: Supplementary file 1 [file TID-19-18-s1.pdf]

tobacconomics
Economic Research Informing
Tobacco Control Policy

# Tobacconomics
# Cigarette Tax Scorecard

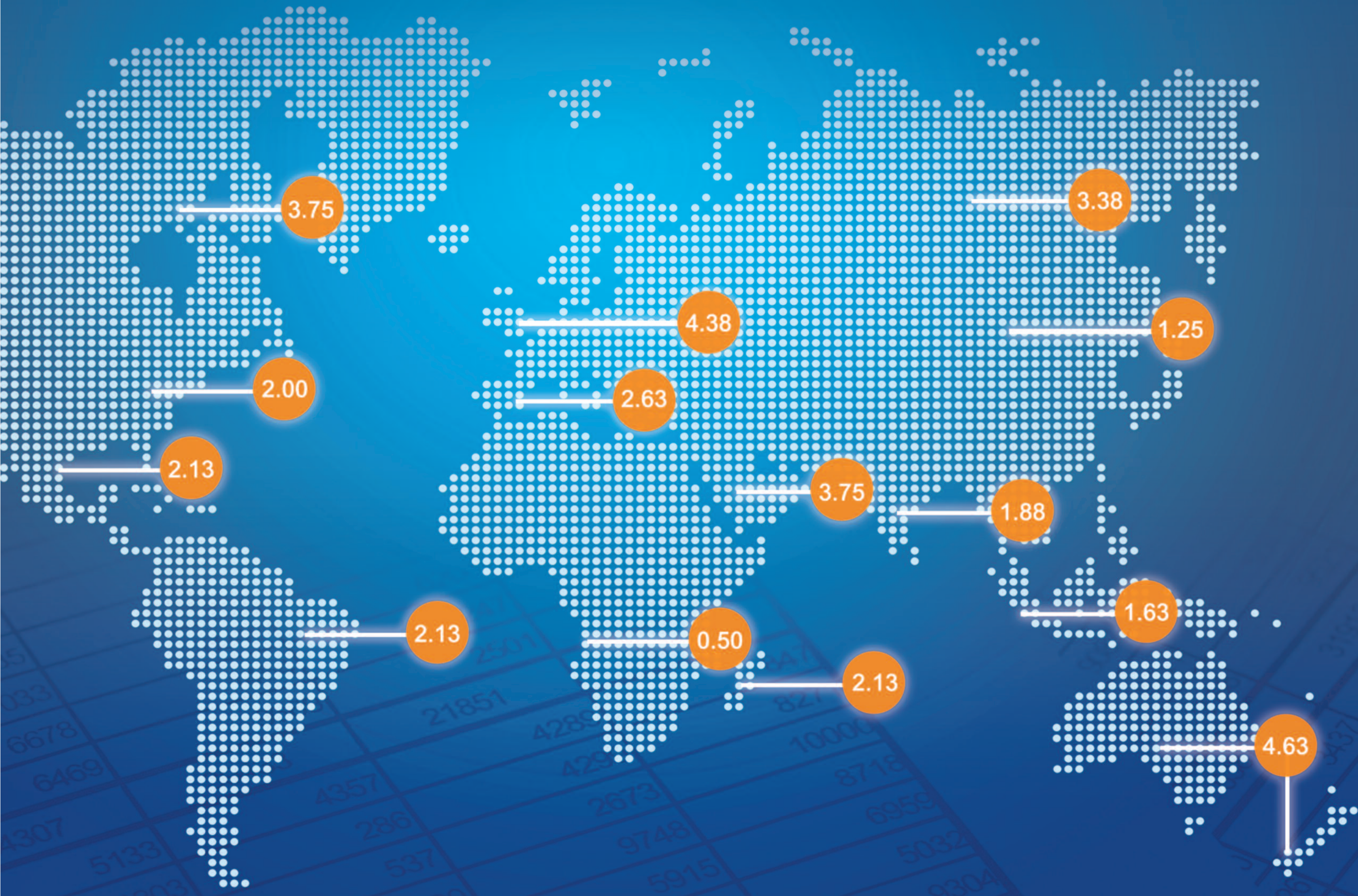

**Suggested Citation:** Chaloupka, F., Drope, J., Siu, E., Vulovic, V., Stoklosa, M., Mirza, M., Rodriguez-Iglesias, G., & Lee, H. Tobacconomics cigarette tax scorecard. Chicago, IL: Health Policy Center, Institute for Health Research and Policy, University of Illinois Chicago, 2020. www.tobacconomics.org

**Authors:** This report was written by the Tobacconomics team: Frank Chaloupka, PhD; Jeff Drope, PhD; Erika Siu, JD LLM; Violeta Vulovic, PhD; Michal Stoklosa, PhD; Maryam Mirza, PhD; Germán Rodriguez-Iglesias, MSc; and Hye Myung Lee, MPH.

**Peer Reviewers:** The report was peer reviewed by Evan Blecher, Economist, Fiscal Policies for Health (TAX), Health Promotion Department, World Health Organization; Alan Fuchs Tarlovsky, Senior Economist, Poverty and Equity Global Practice, The World Bank, Maria Fernanda Gonzalez Icaza, Consultant, Poverty and Equity Global Practice, The World Bank; Rijo M. John, Adjunct Professor, Rajagiri College of Social Sciences, Kochi; Nigar Nargis, Scientific Director, Tobacco Control Policy Research, American Cancer Society; Guillermo Paraje, Professor of Economics, Universidad Adolfo Ibáñez; Anne-Marie Perucic, Economist, Fiscal Policies for Health (TAX), Health Promotion Department, World Health Organization; Maxime Roche, International Consultant, Pan American Health Organization; Rosa Carolina Sandoval, Regional Advisor, Tobacco Control, Pan American Health Organization; Francis Thompson, Tobacco Control Advisor, HealthBridge Foundation of Canada; and Professor Corné van Walbeek, Director of the Research Unit on the Economics of Excisable Products, School of Economics, University of Cape Town.

**About Tobacconomics:** Tobacconomics is a collaboration of leading researchers who have been studying the economics of tobacco control policy for nearly 30 years. The team is dedicated to helping researchers, advocates, and policy makers access the latest and best research about what's working—or not working—to curb tobacco consumption and its economic impacts. As a program of the University of Illinois Chicago, Tobacconomics is not affiliated with any tobacco manufacturer. Visit **www.tobacconomics.org** or follow us on Twitter **www.twitter.com/tobacconomics**.

This Scorecard was funded by Bloomberg Philanthropies. The University of Illinois Chicago (UIC) is a partner of the Bloomberg Initiative to Reduce Tobacco Use. The views expressed in this document cannot be attributed to, nor do they represent, the views of UIC, the Institute for Health Research and Policy, or Bloomberg Philanthropies.

For any comments or questions about this Scorecard, please email us at info@tobacconomics.org.
We very much look forward to hearing from you.

Copyright © 2020 by Tobacconomics. All Rights Reserved.

Cover Design, Interior Design, and Formatting: Alamini Creative Group
Editing: Alison Goldstein, MPH

## Acknowledgments

The authors would like to thank the following individuals for their input on the Scorecard: Johanna Birckmayer, Maria Carmona, Joanna Cohen, Gan Quan, Chris Lane, and Kevin Welding.

# Table of Contents


Executive Summary 2

I. Introduction 3

II. Cigarette Tax Scorecard – Overall Scores 9

III. Cigarette Price 13

IV. Change in Cigarette Affordability 15

V. Tax Share 17

VI. Excise Tax Structure 20

VII. Discussion 23

References 25

Appendices 26


## List of Figures


Figure 1. Overall cigarette tax scores, 2018 10

Figure 2. Cigarette price scores, 2018 16

Figure 3. Change in affordability scores, 2018 16

Figure 4. Tax share scores, 2018 18

Figure 5. Cigarette excise tax structure scores, 2018 21


## List of Tables


Table 1. Overall cigarette tax scores, 2018 10

Table 2. Overall cigarette tax scores, globally and by WHO region, 2018 12

Table 3. Overall cigarette tax scores, globally and by World Bank income group, 2018 12

Table 4. Average cigarette price ($Intl PPP) and average price score, globally and by WHO region, 2018 14

Table 5. Average cigarette price ($Intl PPP) and average price score, globally and by World Bank income group, 2018 14

Table 6. Average annual cigarette affordability change and affordability change score, globally and by WHO region, 2018 16

Table 7. Average annual cigarette affordability change and affordability change score, globally and by World Bank income group, 2018 16

Table 8. Total tax shares, excise tax shares, and tax share scores, globally and by WHO region, 2018 18

Table 9. Total tax shares, excise tax shares, and tax share scores, globally and by World Bank income group, 2018 18

Table 10. Average tax structure scores, globally and by WHO region, 2018 22

Table 11. Average tax structure scores, globally and by World Bank income group, 2018 22

Appendix Table 1. Overall cigarette tax scores, 2018 26

Appendix Table 2-A. Overall and component cigarette tax scores, 2018 29

Appendix Table 2-B. Overall and component cigarette tax scores, 2016 34

Appendix Table 2-C. Overall and component cigarette tax scores, 2014 39

Appendix Table 3. Overall cigarette tax scores, 2014, 2016, and 2018 44

# *Executive Summary*

The global health and economic crises caused by the COVID-19 pandemic have had a devastating impact on government budgets. Increasing tobacco taxes provides a logical first step for governments to raise much needed revenue for economic recovery while promoting public health. Tobacco use—a slow-moving pandemic in itself—accounts for more than eight million deaths each year and about 13 percent of all deaths, costing the world's economies more than US$ 1.4 trillion in health care expenditures and lost productivity. Most of these deaths and economic losses occur in low- and middle-income countries. The single most effective way to reduce the health and economic devastation caused by tobacco use is to significantly increase tobacco taxes and prices. The best way to do this is through a uniform specific excise tax that comprises at least 70 percent of the retail price and is automatically updated to stay ahead of inflation and income growth.

The Tobacconomics Cigarette Tax Scorecard scores cigarette tax policy performance in more than 170 countries on a five-point scale using data from the World Health Organization's biennial Report on the Global Tobacco Epidemic, providing policy makers with an actionable assessment of their country's cigarette tax policy.

**The Scorecard shows that overall, most countries are failing to effectively tax cigarettes:**

Nearly **half scored less than two out of the maximum five points**, and there has been little improvement over the past six years.

The global average score **rose only slightly from 1.85 in 2014 to 2.07 in 2018**. Although overall scores improved in 89 countries, **they became worse in 43 countries**.

**This failure also represents an opportunity: there is considerable untapped potential for cigarette tax increases to raise much needed revenue for a post-COVID-19 recovery and, importantly, save lives and promote a healthy and productive workforce.**

The top-performing countries in this assessment are Australia and New Zealand, scoring the highest at 4.63, which reflects their high, uniform specific cigarette excise taxes with regular increases that have significantly reduced the affordability of cigarettes. The highest-performing region is Europe, with an average score of 2.79. Nevertheless, this is just over half of the possible score of 5.0. Higher-income countries generally have higher taxes and prices and more effective tax structures than lower-income countries.

The countries with the greatest improvement in cigarette tax policy are Bahrain (an overall three-point improvement), Saudi Arabia (+2.75), the United Arab Emirates (+2.75), Kyrgyzstan (+2.50), and the Philippines (+2.50). The improvements in Bahrain, Saudi Arabia, and the United Arab Emirates reflect the introduction of significant cigarette excise taxes, while those in Kyrgyzstan and the Philippines result from the simplification of previously complicated tiered cigarette excise tax structures accompanied by large tax increases.

It is our hope that this Scorecard stimulates greater awareness of the need for more effective tobacco tax policies and motivates policy makers to significantly raise tobacco taxes in order to increase the price of tobacco products to make them less affordable and, ultimately, to reduce tobacco use globally.

# I Introduction

Tobacco use is the leading cause of preventable deaths globally, accounting for more than eight million deaths each year, about 13 percent of all deaths. The vast majority of these deaths occur in low- and middle-income countries (LMICs). Annually, tobacco use costs the world's economies more than US$ 1.4 trillion in health care expenditures and lost productivity. Reducing this health and economic toll is achievable through the implementation of evidence-based, cost-effective policies including smoke-free air laws; prominent graphic warning labels on packaging; bans on tobacco company advertising, promotion, and sponsorship; mass media public education campaigns; and support for cessation efforts. These policies work, but the single most effective way to reduce the health and economic devastation caused by tobacco use is to significantly increase tobacco taxes and prices.

Extensive guidance on best practices in tobacco taxation has been developed by the World Health Organization (WHO), Parties to the WHO's Framework Convention on Tobacco Control (FCTC), the World Bank, and academics and researchers worldwide. The Tobacconomics Cigarette Tax Scorecard incorporates this guidance into a five-point rating system to assess countries' cigarette tax policies based on four established best practices for cigarette taxation. The focus is on taxation of cigarettes, given that they are by far the most widely consumed tobacco product globally and given the availability of comparable data for most countries.

## Why Taxes on Tobacco?

Raising taxes on tobacco works. Evidence from around the world shows that higher taxes lead to higher prices and that these higher prices decrease overall tobacco use, lead current users to quit, prevent young people from initiating tobacco use, and reduce the negative health and economic consequences of tobacco use.

Tobacco tax increases have the greatest impact in reducing tobacco use among vulnerable populations, including young people and low-income populations. Tobacco use among young people is more sensitive to price increases than tobacco use among adults, which is particularly important given that nearly all tobacco users start during adolescence or as young adults. Similarly, low-income tobacco users are more responsive to tax and price increases than higher-income groups in addition to being more susceptible to the damaging health impacts of tobacco use because they often lack access to health care and services and/or are more likely to have other serious health problems. Faced with higher taxes and prices, these users are more likely to quit or reduce their tobacco use.

Increasing tobacco taxes generates new government revenues. Despite the reductions in tobacco use that follow tax increases, country experiences across the globe show that significant tobacco tax increases lead to increases in tobacco tax revenues. This happens because the reductions in tobacco use are less than the increase in price, given the addictive nature of the nicotine in tobacco products. The increases in government revenue can be used to fund public health and other sustainable development priorities. The WHO estimates, for example, that a cigarette tax increase of US$ 1.00 per pack would have raised between US$ 178 and 219 billion in 2018 (Goodchild et al., 2020).

Not surprisingly, a majority of the public, including many smokers, supports tax increases on tobacco products. In fact, most smokers regret ever starting and many try to quit each year, so it is not surprising that a significant proportion of smokers supports tobacco tax increases. Support for tobacco tax increases is even higher when revenues are used to fund tobacco control and other health promotion efforts. When revenues are targeted to strengthen tobacco control, tobacco use declines further.

## Why this Scorecard?

Despite considerable evidence supporting higher tobacco excise taxes and established guidance from international organizations, policy makers—particularly in LMICs—have been slow to adopt these policies. This failure is largely driven by strong opposition from the tobacco industry and its allies, who raise concerns about potential negative economic consequences, suggesting that tax increases lead to increased illicit trade and lost employment and that they are regressive. These concerns are often false or exaggerated and do not justify inaction. Nevertheless, this opposition has slowed or stopped the successful implementation of tobacco taxation as both a viable public health strategy and a way to mobilize much needed government revenue.

**This Cigarette Tax Scorecard aims to comprehensively assess the current status of cigarette taxation across countries by synthesizing established best practices into a five-point grading system, allowing policy makers to easily evaluate the effectiveness of their country's current cigarette tax policy while understanding the specific areas of improvement needed for more effective taxation. Policy makers can also see progress over time and compare their government's performance to others'.**

## Established Best Practices

This Cigarette Tax Scorecard assesses countries' cigarette tax systems with respect to their consistency with the WHO FCTC Article 6 Guidelines, the *WHO Technical Manual on Tobacco Tax Administration*, the World Bank reports *Tobacco Tax Reform: At the Crossroads of Health and Development* and *Curbing the Epidemic: Governments and the Economics of Tobacco Control*, the U.S. National Cancer Institute (NCI) – WHO Monograph 21: *The Economics of Tobacco and Tobacco Control*, and other seminal research on effective tobacco taxation.

**WHO FCTC Article 6 and Article 6 Guidelines (2014)** The WHO FCTC, the world's first public health treaty under the auspices of the WHO, entered into force in February 2005 and currently has 182 Parties, covering 90 percent of the world population. While acknowledging tax sovereignty, Article 6 of the treaty calls on Parties to use price and tax measures to reduce the demand for tobacco products, especially among youths (WHO, 2003). Guidelines on Article 6 were adopted by the Conference of the Parties and are based on evidence, best practices, and experiences of the Parties that have successfully implemented tax and price measures to reduce tobacco consumption (WHO, 2014).

**WHO Technical Manual on Tobacco Tax Administration (2010)** This technical manual identifies best practices for tobacco taxation, shares governments' existing approaches to tobacco taxation, discusses barriers to using tobacco taxes to achieve health and revenue objectives, and provides case studies of effective tobacco tax administration (WHO, 2010).

**World Bank Tobacco Tax Reform (2017) and Curbing the Epidemic reports (1999)** These reports examine economic questions and policy options for tobacco taxation and other tobacco control measures, analyze global trends in tobacco use, and assess the consequences of tobacco control for health, economies, and individuals. Both reports draw on the existing global evidence, particularly evidence from low- and middle-income countries (World Bank, 2017; Jha & Chaloupka, 1999).

**NCI-WHO Monograph 21 (2018)** The Monograph systematically examines the extensive global research and evidence base surrounding the economics of tobacco control (NCI & WHO, 2018). Chapter 4 of the Monograph discusses models of the demand for tobacco products, evidence of the impact of taxes and prices on the demand for tobacco products, and the effect of factors such as age and gender on sensitivity to changes in the price of tobacco products. Chapter 5 of the Monograph reviews the evidence on the design and administration of tobacco taxes.

## Primary Data Source

The five-point grading system is derived from the data in the tax/price-related appendices of the biennial WHO Report on the Global Tobacco Epidemic (RGTE). The report monitors the status of the tobacco epidemic and the most effective and cost-effective government interventions—both price and non-price measures—for reducing tobacco consumption.

## The Four Grading Components

Synthesizing these guidelines and best practices, the Tobacconomics Cigarette Tax Scorecard uses a five-point index based on four key components outlined below: cigarette price, changes in the affordability of cigarettes over time, the share of taxes in retail cigarette prices, and the structure of cigarette taxes. Each of the four components is scored using a five-point index, with the total score reflecting an average of the four component scores.

### *Component 1: Cigarette Price*

Price is a key determinant of tobacco use. As the price of a product increases, consumers use less of it; and as the price of a product decreases, consumers use more of it. Economists look at the relationship between prices and consumption through a measure called the "price elasticity of demand," or the percentage change in consumption resulting from a one-percent change in price. If the change in consumption is less than proportional to the change in price, demand for the product is defined as price inelastic; if the change in consumption is more than proportional to the change in price, the product's demand is price elastic.

While higher prices reduce consumption, cigarettes are relatively price inelastic: an increase in price will result in a less-than-proportional decline in consumption. Therefore, price must be sufficiently high to reduce consumption enough to generate clear public health benefits. Any metric that compares prices across countries must be based on a measure that takes consumers' purchasing power into account; in this Scorecard, purchasing power parity (PPP) adjusted prices are used. These aspects are reflected in the guiding documents in the following ways:

- The FCTC Article 6 Guidelines state that increases in prices reduce tobacco use (2014).
- The WHO tobacco tax manual states that price needs to be sufficiently high (2010).
- The World Bank *Tobacco Tax Reform* report highlights the importance of large price increases in reducing cigarette smoking (2017).
- The NCI-WHO Monograph describes the extensive evidence on the price elasticity of cigarette demand (2018).

Based on the extensive research evidence and these recommendations, the Tobacconomics Tax Scorecard gives the highest score to a PPP-adjusted price of ten international dollars or higher in 2018, adjusted for inflation, for a pack of 20 of the most-sold brand of cigarettes.

## *Component 2: Changes in Cigarette Affordability*

Income, as well as price, influences demand. For most goods and services, an increase in income causes an increase in demand and, thus, an increase in consumption. When additional income is spent on goods and services like health and education, it enhances human well-being. In contrast, additional income spent on harmful goods like tobacco that also have significant negative externalities can lead to considerable economic and health-related costs for individuals and societies.

Rapid economic growth resulting in increases in income can offset increases in taxes and prices and limit their impact on consumption. Simultaneously considering the impact of price and income introduces the concept of affordability, which is broadly defined as the ratio of price to income. Research demonstrates that increasing affordability of cigarettes leads to an increase in consumption, while decreasing affordability reduces consumption. Increases in cigarette taxes and prices must be high enough to reduce cigarette affordability and impact use.

- The FCTC Article 6 Guidelines emphasize the importance of raising taxes to account for inflation and income growth to reduce affordability (2014).
- The WHO tobacco tax manual recommends raising taxes to reduce affordability (2010).
- The World Bank *Tobacco Tax Reform* report emphasizes the need to “attack affordability” (2017).
- The NCI-WHO Monograph highlights research showing the importance of reducing affordability for reducing tobacco use (2018).

Based on the research evidence and these recommendations, the Tobacconomics Tax Scorecard gives the highest score for a statistically significant annual average change in affordability of 7.5 percent or higher between 2012 and 2018 that is the result of at least one excise tax increase during that period.

## *Component 3: Tax Shares*

The share of tax in retail price is a key tax performance measure. Higher tax shares generally result in higher retail prices and reductions in tobacco use. The higher the tax share, the more the government gains in revenue. Tax shares should be high enough to allow governments to gain revenue from the price increase while also reducing tobacco use. If a price increase results from industry price increases alone—although consumption will fall—the revenues will go to the tobacco industry.

The share of taxes in cigarette prices has been the focus of multiple recommendations and has been the most widely used metric for assessing the strength of tobacco tax systems globally. These recommendations have varied with respect to which taxes are included and what the recommended share of tax should be. Some focus on the share of excise taxes in retail cigarette prices, while others include additional taxes such as import duties, general sales taxes, or value added taxes. Despite these differences, all recommend that taxes should account for most of retail prices.

- The WHO tobacco tax manual recommends that excise taxes account for at least 70 percent of retail price; FCTC Article 6 Guidelines cite this in a footnote when discussing appropriate tax levels (WHO, 2010 & 2014).
- The WHO reports on the global tobacco epidemic use 75 percent or more of total tax share to reflect a high level of achievement (2015).

- The World Bank's *Curbing the Epidemic* report recommends that taxes account for two-thirds to four-fifths of retail price (Jha & Chaloupka, 1999).

Based on these recommendations, this Tobacconomics Tax Scorecard component gives the highest scores for a 70 percent or greater excise share and a 75 percent or higher total tax share, averaging the separate scores for each of the two tax shares to create a single tax share score.

## *Component 4: Tax Structure*

Tax structures are critical in ensuring that tax increases reduce tobacco use and increase government revenues. First, the tax structure determines the object of the tax. Excise taxes on tobacco products are more effective in reducing tobacco use than other generally applied taxes on goods and services because these taxes drive tobacco product prices higher relative to other goods and services. Second, the type of excise tax is important. An excise tax may be an ad valorem tax, where the rate is applied to a determined value of the product, or it may be a specific tax applied to a defined unit of the product. Most countries apply either a specific or an ad valorem excise tax on cigarettes, while some use a combination of the two (a mixed system). Some countries relying on ad valorem excise taxes and others that use a mixed system specify a minimum specific tax, which helps to keep prices for discount brands higher.

Additionally, the base on which the tax is assessed has a direct impact on the final price and revenues generated by the tax. For example, an ad valorem tax may be assessed on the retail price, which reflects the costs of production and distribution, or the ex-factory price, which reflects only the costs of production. A specific tax, however, is assessed on the unit of product and is easier to administer. Some countries define multiple tax tiers for cigarettes, often classified by price or other distinctive characteristics. Finally, specific excise taxes need to be updated regularly in order to keep the real value of the tax from falling and to prevent affordability from increasing.

Recommendations:

- The WHO FCTC Article 6 Guidelines recommend a uniform specific tax structure or mixed structure of ad valorem and specific taxation that relies more on the specific component and a minimum specific tax (2014).
- The WHO tobacco tax manual emphasizes specific excise taxes and uniform tax structures (2010).
- The World Bank *Tobacco Tax Reform* report recommends taxing by quantity to minimize downtrading in response to cigarette tax increases (2017).
- Research evidence in the NCI-WHO Monograph highlights the benefits of uniform tax structures and tax structures that emphasize specific taxes (2018).

Based on the research evidence and these recommendations, this component of the Tobacconomics Cigarette Tax Scorecard gives the highest score for either: (1) a uniform specific excise tax that is automatically adjusted; or (2) a mixed excise tax with an emphasis on the specific component in addition to a minimum tax, an automatic adjustment to the specific tax component, and the use of the retail price as the base for the ad valorem tax component.

## Road Map to the Scorecard

This first edition of the Scorecard uses the most recent 2019 *WHO Report on the Global Tobacco Epidemic* (RGTE) (which reports 2018 data). The next edition of the Scorecard will be released in 2021 using the 2021 RGTE (2020 data); comparable scores are constructed for 2016 and 2014, using data in the 2017 and 2015 RGTE respectively, in order to assess changes over time in cigarette tax systems. Going forward, the Scorecard will be released biennially using the latest RGTE data. Additional materials based on this Scorecard will be developed. What follows is a narrative report that briefly describes the overall scoring results and the scoring for each of the four components. Appendices provide the country-by-country overall scores as well as scores for each grading component. Data presented by region reflect the seven regional groupings defined by WHO (African region – AFR; region of the Americas – AMR; Eastern Mediterranean region – EMR; European region – EUR; South-East Asia region – SEAR; and Western Pacific region – WPR), while data presented by income level reflect the income categories defined by the World Bank.

# II Cigarette Tax Scorecard – Overall Scores

The overall cigarette tax scores for 2018 are shown in Figure 1 and Table 1 for the 174 countries with available data for each of the four components. This composite score is constructed as the simple average of the scores on each of the four key components: cigarette price, change in cigarette affordability, share of taxes in cigarette prices, and cigarette tax structure. The overall score can range from a low of zero, for countries that score zero on each component, to a high of five, for countries that receive the highest score on each component. Scores for each of the four components are discussed below.

In 2018, only four countries received a score of four or higher, led by Australia and New Zealand, with scores of 4.63, and followed by Ecuador and the United Kingdom, with scores of 4.38. The high scores in Australia and New Zealand reflect their very high, uniform specific cigarette excise taxes that result in very high cigarette prices, as well as the regular increases in cigarette taxes in recent years that have led to significant reductions in the affordability of cigarettes. Beginning in 2013, Australia has increased its cigarette excise tax by 12.5 percent each year, in addition to semiannual increases tied to inflation (prior to 2014) or wage growth (since 2014). Similarly, beginning in 2010, New Zealand has raised its cigarette tax by at least ten percent plus inflation in January of each year. Ecuador has a uniform specific tax that was doubled in 2018, resulting in high cigarette prices and a sharp reduction in affordability. The United Kingdom is the only country among the top four that uses a mixed cigarette excise tax system; its mixed system includes a significant specific tax component that is automatically increased each year and an ad valorem component that is levied based on retail cigarette prices, with additional increases in taxes beyond inflation that have led to a significant reduction in the affordability of cigarettes.

At the other end of the spectrum, Iraq is the only country with a score of zero in 2018, reflecting its lack of a cigarette excise tax and minimal other taxes, resulting in very inexpensive cigarettes and affordability that has not significantly changed over time. Afghanistan and Libya do only marginally better, with overall scores of 0.25 in 2018. Neither country has an excise tax on cigarettes. Afghanistan has seen some reduction in the affordability of cigarettes in recent years, but not as the result of an increase in taxes on cigarettes. Libya gets some credit for having cigarette prices a bit above the lowest levels in the world.

As shown in Table 2, the European region is doing better than other regions, with an average score of 2.79; nevertheless, this is just over half of the possible 5.0 score for countries performing at the highest level across all components. The relatively high score in the European region reflects stronger tax structures and higher taxes and prices that result from the European Union's tobacco tax directive, with which the 28 member countries (including the United Kingdom in 2018) are required to comply, as well as the implementation of similar taxes in countries aspiring to join the union. The African region is doing the worst, with an average score of 1.36.

Table 3 shows the scores by World Bank income category. There is a clear relationship between overall scores and income, with average scores rising with income. Higher-income countries generally have higher taxes and prices and stronger tax structures than lower-income countries. That said, there are many country-specific exceptions to this for the individual components (see Appendix).

There has been some improvement in the overall scores over time, with the global average score rising from 1.85 in 2014 to 2.07 in 2018. Among the 165 countries for which scores could be computed in both years, overall scores have improved in 89 countries, stayed the same in 33 countries, and worsened in 43 countries. Scores improved the most in Bahrain (an overall three-point improvement), Saudi Arabia (+2.75), the United Arab Emirates (+2.75), Kyrgyzstan (+2.50), and the Philippines (+2.50). The improvements in Bahrain, Saudi Arabia, and the United Arab Emirates reflect the introduction of substantial cigarette excise taxes, after previously relying on import duties, while those in Kyrgyzstan and the Philippines resulted from the simplification of previously complicated tiered cigarette excise tax structures accompanied by large tax increases. In most countries where scores fell over time, the drop in scores is most often explained by a failure to maintain declines in cigarette affordability.

**Figure 1** Overall cigarette tax scores, 2018

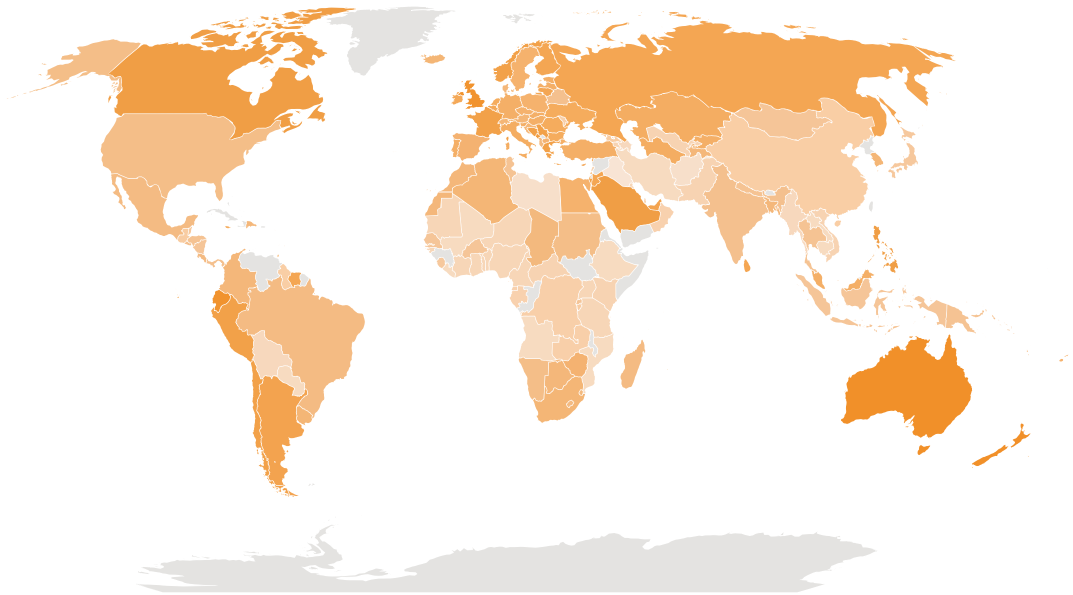


Note: Countries in gray lack available data on this measure.

**Table 1** Overall cigarette tax scores, 2018, from lowest to highest, by score

| Score < 1.0 N=41 | 1.0 ≤ Score < 2.0 N=41 | 2.0 ≤ Score < 3.0 N=48 | 3.0 ≤ Score < 4.0 N=40 | Score ≥ 4.0 N=4 |
|---|---|---|---|---|
| Iraq | Equatorial Guinea | Namibia | Italy | Ecuador |
| Afghanistan | Gabon | Sudan | Kazakhstan | United Kingdom |
| Libya | Oman | United States of America | Turkmenistan | Australia |
| Angola | Saint Kitts and Nevis | Barbados | Bulgaria | New Zealand |
| Antigua and Barbuda | Tuvalu | Brazil | Gambia | |
| Cambodia | Comoros | Costa Rica | Jamaica | |
| Ethiopia | Congo | Lesotho | Malta | |

| Score < 1.0<br>N=41 | 1.0 ≤ Score < 2.0<br>N=41 | 2.0 ≤ Score < 3.0<br>N=48 | 3.0 ≤ Score < 4.0<br>N=40 | Score ≥ 4.0<br>N=4 |
|---|---|---|---|---|
| Iran (Islamic Republic of) | Grenada | Madagascar | Netherlands | |
| Lao People's Democratic Republic | Republic of Moldova | Mexico | Romania | |
| Liberia | Zambia | Vanuatu | Samoa | |
| Mali | China | Albania | Seychelles | |
| Marshall Islands | Dominica | Chad | Ukraine | |
| Mozambique | Guyana | Eswatini | Belgium | |
| Paraguay | Maldives | Bangladesh | Ireland | |
| Azerbaijan | Sao Tome and Principe | Botswana | Mauritius | |
| Bolivia (Plurinational State of) | Burundi | Colombia | Singapore | |
| Democratic Republic of the Congo | Georgia | Denmark | Trinidad and Tobago | |
| Myanmar | Guatemala | Dominican Republic | Finland | |
| Benin | Belize | Luxembourg | Jordan | |
| Cabo Verde | Japan | Morocco | Lithuania | |
| Cameroon | Nauru | Tajikistan | Russian Federation | |
| Côte d'Ivoire | Saint Vincent and the Grenadines | Algeria | Sri Lanka | |
| Ghana | Sierra Leone | Austria | Suriname | |
| Guinea-Bissau | Burkina Faso | Croatia | Argentina | |
| Kuwait | Indonesia | Iceland | United Arab Emirates | |
| Mauritania | Mongolia | Republic of Korea | Bosnia and Herzegovina | |
| Micronesia (Federated States of) | Nicaragua | South Africa | Chile | |
| Niger | Rwanda | Switzerland | France | |
| Nigeria | Senegal | Uruguay | Greece | |
| Solomon Islands | Honduras | North Macedonia | Israel | |
| Togo | Kiribati | Spain | Norway | |
| Uganda | Nepal | Zimbabwe | Peru | |
| United Republic of Tanzania | Papua New Guinea | Czechia | Serbia | |
| Armenia | Thailand | Egypt | Tonga | |
| Central African Republic | Timor-Leste | Fiji | Bahrain | |
| Kenya | Tunisia | Poland | Canada | |
| Lebanon | Belarus | Sweden | Philippines | |
| Pakistan | El Salvador | Cyprus | Saudi Arabia | |
| Qatar | India | Estonia | Montenegro | |
| Uzbekistan | Panama | Germany | Palau | |
| Viet Nam | Saint Lucia | Hungary | | |
| | | Kyrgyzstan | | |
| | | Latvia | | |
| | | Malaysia | | |
| | | Portugal | | |
| | | Slovakia | | |
| | | Slovenia | | |
| | | Turkey | | |

Note: Countries in each column are listed in order of their scores, from lowest to highest, and alphabetically when scores are identical.

**Table 2** Overall cigarette tax scores, globally and by WHO region, 2018

| Region | AFR | AMR | EMR | EUR | SEAR | WPR | Global |
|---|---|---|---|---|---|---|---|
| Score | 1.36 | 2.13 | 1.68 | 2.79 | 1.82 | 2.14 | 2.07 |

**Table 3** Overall cigarette tax scores, globally and by World Bank income group, 2018

| Income Group | Low | Lower-Middle | Upper-Middle | High | Global |
|---|---|---|---|---|---|
| Score | 1.26 | 1.51 | 2.13 | 2.85 | 2.07 |

# III *Cigarette Price*

Given the extensive evidence on the impact of prices on smoking behavior, the price of cigarettes is a key indicator for the performance of a country's tobacco tax system. This Scorecard component is based on the price of a 20-cigarette pack of the most-sold brand in international dollars, adjusted for purchasing power parity (PPP). Based on the prices reported for 2018, scores are based on the following:

**Scoring – Cigarette Price:**

5: Price ≥ 10.0 Intl$ PPP

4: 8.0 ≤ price < 10.0

3: 6.0 ≤ price < 8.0

2: 4.0 ≤ price < 6.0

1: 2.0 ≤ price < 4.0

0: Price < 2.0 Intl$ PPP

Figure 2 shows the cigarette price scores for 2018. Among the 174 countries with available data, 19 countries received the highest score of five, led by Sri Lanka ($22.17), Turkmenistan ($18.81), Saudi Arabia ($17.68), Singapore ($16.87), and Jamaica ($16.59). Twelve countries received a score of zero, with the lowest prices in Paraguay ($0.80), Iraq ($1.24), Democratic Republic of the Congo ($1.28), Cambodia ($1.42), and Afghanistan ($1.50). As shown in Table 4, average cigarette prices and scores were higher in the European, South-East Asia, and Western Pacific regions and lowest in the African region. Average prices and price scores rise with income, as shown in Table 5.

Cigarette price scores, adjusted for inflation, have risen over time, from an average of 1.73 in 2014 to 2.35 in 2018. The number of countries receiving the highest score has nearly tripled from 2014 (7) to 2018 (19), while the number of countries receiving the lowest score has more than halved (from 26 in 2014 to 12 in 2018).

## Figure 2 Cigarette price scores, 2018

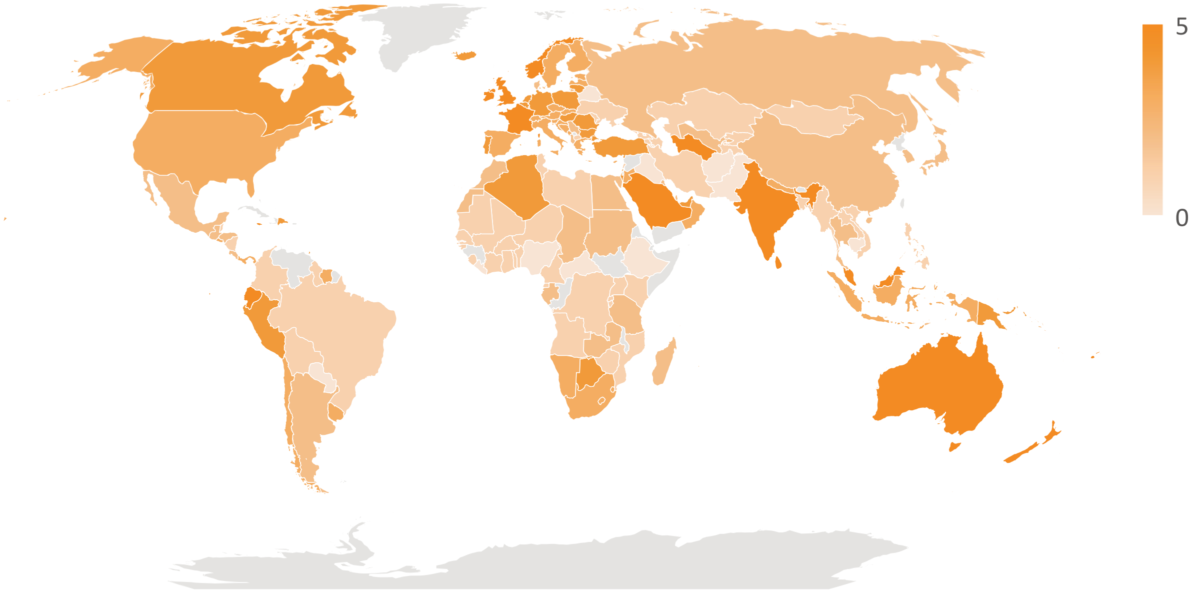


Note: Based on 174 countries in 2018; countries in gray lack available data on this measure.

**Table 4** Average cigarette price ($Intl PPP) and average price score, globally and by WHO region, 2018

| Region | AFR | AMR | EMR | EUR | SEAR | WPR | Global |
|---|---|---|---|---|---|---|---|
| Price | $4.03 | $5.95 | $5.62 | $7.31 | $7.43 | $7.12 | $6.07 |
| Score | 1.49 | 2.39 | 2.00 | 2.94 | 2.78 | 2.71 | 2.35 |

**Table 5** Average cigarette price ($Intl PPP) and average price score, globally and by World Bank income group, 2018

| Income Group | Low | Lower-Middle | Upper-Middle | High | Global |
|---|---|---|---|---|---|
| Price | $2.98 | $4.48 | $6.32 | $8.57 | $6.07 |
| Score | 1.04 | 1.58 | 2.47 | 3.47 | 2.35 |

# IV Change in Cigarette Affordability

In countries where incomes have risen rapidly, it has become increasingly clear that cigarette taxes need to increase enough to raise prices by more than increases in income in order to reduce the affordability of cigarettes. This is captured by the second component of the Tobacconomics Cigarette Tax Scorecard, which assesses changes in cigarette affordability over a six-year period. Affordability is defined as the percentage of per capita GDP required to purchase 2000 cigarettes of the most-sold brand, with an increase in this measure implying that cigarettes are becoming less affordable over time. In order to avoid giving credit to countries where affordability has fallen due to reduced incomes or higher industry prices, higher scores are given to countries where the reduction in affordability has at least partly resulted from a cigarette excise tax increase. Statistical significance of the change in affordability is based on the approach used in the WHO's RGTE, which uses a simple model that regresses the natural logarithm of the affordability measure on a year variable. The 2018 scores for this component are based on statistically significant changes in the affordability of the most-sold brand of cigarettes between 2012 and 2018, as follows:

**Scoring – Change in Affordability:**

5: 7.5% average annual change or higher

4: 5.0% ≤ average annual change < 7.5%

3: 2.5% ≤ average annual change < 5.0%

2: Average annual change < 2.5%

1: Reduced affordability, but no excise tax increase

0: Increased affordability or no statistically significant change

Figure 3 shows the scores for the changes in cigarette affordability between 2012 and 2018. Among the 186 countries with available data, 23 countries received the highest score of five, led by Saudi Arabia (average annual reduction of 19.87 percent), Algeria (18.50 percent), the Gambia (16.52 percent), the Philippines (15.69 percent), and the United Arab Emirates (15.56 percent). In contrast, most countries—123 of the 186—received a score of zero because they saw either no statistically significant change in affordability over time (95 countries) or a significant increase in affordability (28 countries). Eleven countries saw a significant decline in cigarette affordability between 2012 and 2018 but did not increase their cigarette excise tax during this period. The scores for the remaining 29 countries were distributed as follows: 4.0 – 11 countries, 3.0 – 13 countries, and 2.0 – five countries.

Table 6 shows the average changes in affordability among countries that have seen significant changes in affordability, by region, as well as the average scores regionally and globally for the affordability component of the Scorecard; in computing these averages, countries with non-significant changes in affordability were assigned a score of zero. The largest declines in affordability have occurred in the Eastern Mediterranean region (average annual decline of 4.80 percent) followed by the South-East Asia and Americas regions. While there have been large reductions in affordability in some African countries, the region has seen the slowest average drop in affordability among countries with significant changes and has the lowest average

score among the regions. As shown in Table 7, lower-middle-income countries score worst on the affordability measure, with a score that is less than half that for other income groups, while the average scores for other income groups are similar. The lower scores for lower-middle-income countries are at least in part attributable to the relatively greater increases in income in these countries.

Overall, there has been a slight drop in the cigarette affordability scores between 2014 (a global average score of 1.25) and 2018 (global average of 1.18). An increase in countries with the highest score of five (from 16 countries in 2014 to 23 countries in 2018) has been offset by a rise in the number of countries scoring zero (from 114 in 2014 to 123 in 2018).

## Figure 3 Change in affordability scores, 2018

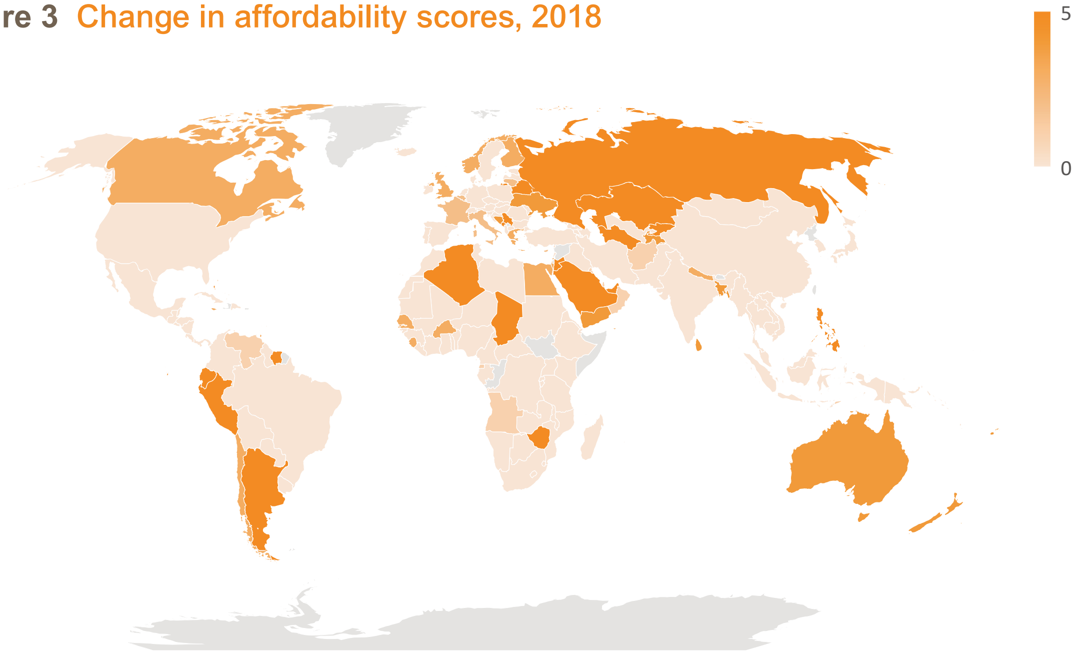


Note: Countries in gray lack available data on this measure.

## Table 6 Average annual cigarette affordability change and affordability change score, globally and by WHO region, 2018

| Region | AFR | AMR | EMR | EUR | SEAR | WPR | Global |
|---|---|---|---|---|---|---|---|
| **Affordability change** | 1.43% | 2.89% | 4.80% | 1.66% | 4.05% | 1.76% | 2.29% |
| **Score** | 0.70 | 1.18 | 1.55 | 1.40 | 1.33 | 1.24 | 1.18 |

## Table 7 Average annual cigarette affordability change and affordability change score, globally and by World Bank income group, 2018

| Income Group | Low | Lower-Middle | Upper-Middle | High | Global |
|---|---|---|---|---|---|
| **Affordability change** | 2.63% | 0.88% | 3.27% | 2.29% | 2.29% |
| **Score** | 1.28 | 0.62 | 1.40 | 1.35 | 1.18 |

# V Tax Share

The most commonly used metric for assessing the strength of countries' cigarette tax systems has been the share of taxes in retail cigarette prices. Over two decades ago, the World Bank recommended that taxes should account for between two-thirds and four-fifths of cigarette prices. More recently, in its biennial reports on the global tobacco epidemic, WHO describes countries where taxes are at least 75 percent of retail price as the highest achieving countries. Others have focused on the share of excise taxes in retail prices, given that excise taxes are more important in raising the price of cigarettes relative to the prices of other products and, as a result, will have a greater impact on cigarette smoking. Each measure has its own strengths and limitations. For these reasons, the Tobacconomics Cigarette Tax Scorecard tax share component is based on the average of the scores for two tax share indicators – one based on the share of all taxes in cigarette prices and the other focused on the share of excise taxes in prices. The scoring for each is as follows:

**Scoring – Total Tax Share:**

5: 75% total tax share or higher
4: 65% ≤ share < 75%
3: 55% ≤ share < 65%
2: 45% ≤ share < 55%
1: 35% ≤ share < 45%
0: Total tax share < 35%

**Scoring – Excise Tax Share:**

5: 70% excise tax share or higher
4: 60% ≤ share < 70%
3: 50% ≤ share < 60%
2: 40% ≤ share < 50%
1: 30% ≤ share < 40%
0: Excise tax share < 30%

Figure 4 shows the cigarette tax share scores for 2018. Of the 185 countries with available data, only four received the highest score of five: Andorra (79.34 percent total tax share, 75.03 percent excise tax share), Argentina (76.22 percent, 71.20 percent), Egypt (77.19 percent, 77.19 percent), and Mauritius (83.54 percent, 70.50 percent). An additional 34 countries received the highest score for their total tax share, but not for their excise tax share. In contrast, only two countries (Cuba and Palau) received the highest excise tax share score but a lower total tax share score. At the other end of the spectrum, 45 countries received a zero score for their total tax share and 70 received a zero score for their excise tax share, with 44 countries scoring zero for both. As shown in Table 8, tax shares and tax share scores are highest in the European region, largely due to the European Union tobacco tax directive that requires member states to implement relatively high excise taxes on cigarettes. In contrast, tax shares and scores are lowest in the African region. As with cigarette prices, tax shares and tax share scores rise with income, with the average tax share score nearly five times higher in high-income countries than in low-income countries.

There has been little improvement in tax share scores over time, with the global average score rising from 1.91 in 2014 to 2.06 in 2018. Of the 182 countries with data for both 2014 and 2018, most—103—saw no change in their tax share score. Tax share scores increased in 51 countries between 2014 and 2018, led by three-point increases in Colombia, which implemented a significant cigarette excise tax increase in 2017, and three Gulf Cooperation Council countries (Bahrain, Saudi Arabia, and the United Arab Emirates), which introduced new excise taxes on cigarettes. At the same time, tax share scores fell from 2014 to 2018 in 28 countries.

## Figure 4 Tax share scores, 2018

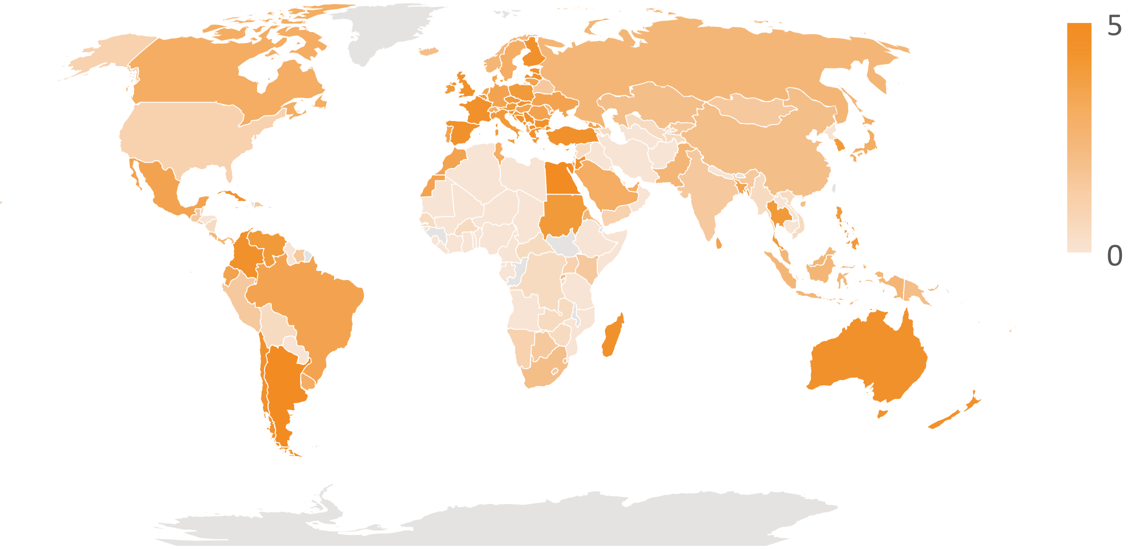


Note: Countries in gray lack available data on this measure.

## Table 8 Total tax shares, excise tax shares, and tax share scores, globally and by WHO region, 2018

| Region | AFR | AMR | EMR | EUR | SEAR | WPR | Global |
|---|---|---|---|---|---|---|---|
| Total tax share | 37.11% | 48.90% | 47.21% | 69.58% | 48.14% | 55.06% | 52.43% |
| Total tax share score | 1.00 | 2.09 | 2.24 | 3.96 | 2.20 | 2.50 | 2.43 |
| Excise tax share | 24.08% | 36.00% | 30.50% | 53.04% | 31.97% | 38.10% | 37.32% |
| Excise tax share score | 0.61 | 1.45 | 1.52 | 2.84 | 1.30 | 1.81 | 1.69 |
| Combined tax share score | 0.81 | 1.77 | 1.88 | 3.40 | 1.75 | 2.15 | 2.06 |

**Table 9** Total tax shares, excise tax shares, and tax share scores, globally and by World Bank income group, 2018

| Income Group | Low | Lower-Middle | Upper-Middle | High | Global |
|---|---|---|---|---|---|
| **Total tax share** | 32.89% | 46.83% | 53.05% | 66.94% | 52.43% |
| **Total tax share score** | 0.83 | 1.82 | 2.43 | 3.78 | 2.43 |
| **Excise tax share** | 19.28% | 34.11% | 35.78% | 51.29% | 37.32% |
| **Excise tax share score** | 0.33 | 1.25 | 1.59 | 2.87 | 1.69 |
| **Combined tax share score** | 0.58 | 1.53 | 2.01 | 3.33 | 2.06 |

# VI Excise Tax Structure

The structure of an excise tax determines its effectiveness in achieving the public health and revenue goals of the tax, with simple, uniform tax structures having greater impact. This component of the Scorecard assesses multiple dimensions of cigarette excise tax structures as follows:

**Scoring – Tax Structure:**

5: A uniform specific tax with an automatic inflation or other adjustment; or a uniform mixed system with greater share of specific tax, with an automatic adjustment for the specific component, the retail price as the base for the ad valorem component, and a minimum specific tax

4: A uniform specific tax or uniform mixed system with a greater share of specific tax, but not other features listed above

3: A uniform mixed system with a greater share of ad valorem tax

2: A uniform ad valorem tax

1: A tiered specific or ad valorem excise tax

0: No excise tax

Figure 5 shows the tax structure scores for 2018. Of the 185 countries with available data, 12 countries received the highest score of five. Of these, seven countries implement a uniform specific cigarette excise tax that is automatically adjusted for inflation or other factors: Australia, Canada, Honduras, New Zealand, Nicaragua, the Philippines, and South Africa. Five countries apply a uniform mixed system with a greater share for the specific tax, an automatic adjustment for the specific tax, a retail price base for the ad valorem tax, and a minimum specific tax: Netherlands, North Macedonia, Romania, Sweden, and the United Kingdom. An additional 65 countries use either a uniform specific tax that is not automatically adjusted or a mixed system with a greater share of specific tax that does not include each of the three features required for the highest score. Twenty-two countries use a uniform mixed system that gives greater weight to the ad valorem component, while 40 apply a uniform ad valorem system. There are 31 countries that use some form of tiered excise tax structure, with rates varying based on price, cigarette length, presence of a filter, cigarette packaging, production type and/or level, and/or other factors. Finally, 15 countries do not levy an excise tax on cigarettes, instead relying on import duties and/or other taxes.

The average tax structure scores by WHO regions are presented in Table 10. The European region is the highest-performing region, in part reflecting the uniform mixed system with a minimum tax mandated by the European Union's tobacco tax directive, but it is followed closely by the region of the Americas. The lowest-scoring regions are South-East Asia and the Eastern Mediterranean. The low score for the South-East Asia region reflects the tiered cigarette excise tax systems implemented in many of the region's countries, including Bangladesh, India, Indonesia, Myanmar, Nepal, Sri Lanka, and Thailand, while the low score for the Eastern Mediterranean region results from the lack of a cigarette excise tax in several countries, including Afghanistan, Iraq, Kuwait, Libya, Qatar, and Somalia. The average scores by World Bank income groups are shown in Table 11. As with the cigarette price and tax share measures, tax structure scores rise with income.

Tax structure scores have changed little over time, rising from a global average score of 2.48 in 2014 to 2.69 in 2018. The vast majority of countries have not changed their tax structures during this period. The most significant changes to tax structure were implemented in Belize and the Philippines. After having no cigarette excise tax, Belize introduced a uniform specific excise, raising its score for this component from zero to four, while the Philippines went from a tiered tax system to uniform specific excise tax with automatic adjustments, raising its score from one to five. An additional 27 countries saw improvements in their tax structure score from 2014 to 2018. In contrast, nine countries saw their tax structure score fall from 2014 to 2018, including Turkmenistan (a change from a uniform specific to a uniform ad valorem tax), Thailand (the replacement of a uniform ad valorem tax with a tiered ad valorem tax based on price), and Kenya (which reinstated a tiered specific tax in 2015).

**Figure 5** Cigarette excise tax structure scores, 2018

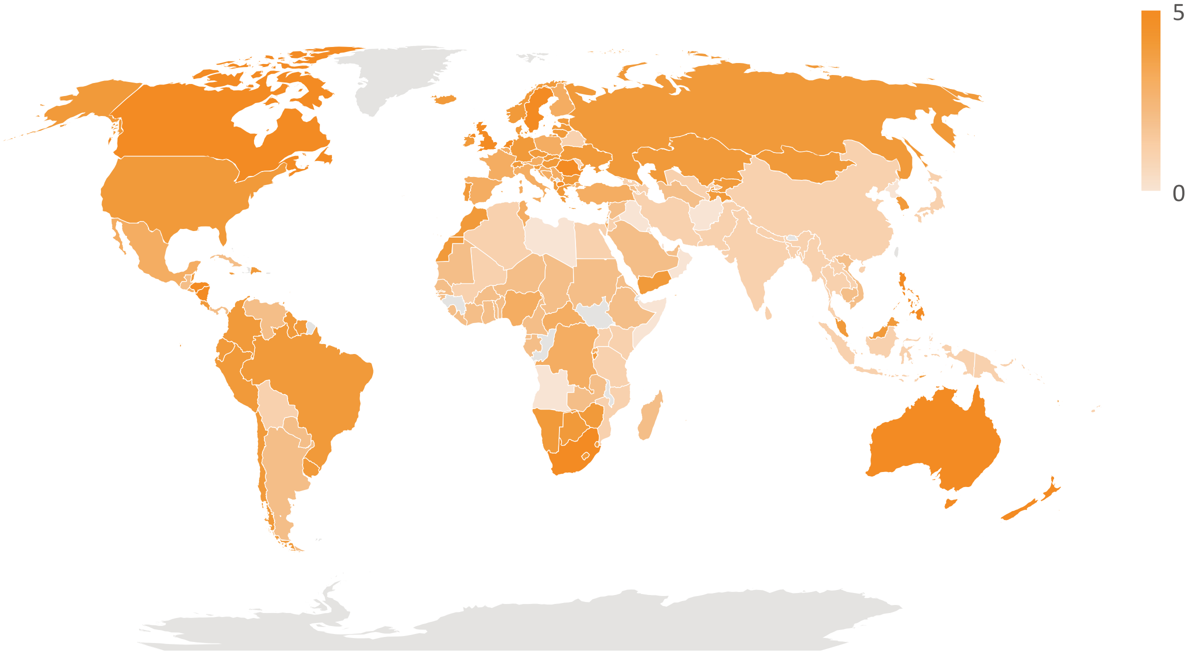


Note: Countries in gray lack available data on this measure.

**Table 10** Average tax structure scores, globally and by WHO region, 2018

| Region | AFR | AMR | EMR | EUR | SEAR | WPR | Global |
|---|---|---|---|---|---|---|---|
| Score | 2.43 | 3.33 | 1.43 | 3.43 | 1.10 | 2.46 | 2.69 |

**Table 11** Average tax structure scores, globally and by World Bank income group, 2018

| Income Group | Low | Lower-Middle | Upper-Middle | High | Global |
|---|---|---|---|---|---|
| Score | 2.03 | 2.36 | 2.64 | 3.35 | 2.69 |

# VII Discussion

This first edition of the Tobacconomics Cigarette Tax Scorecard aims to provide a comprehensive, transparent, objective, and simple approach to assessing the strength of cigarette tax systems globally. It recognizes that a single indicator is insufficient. The most widely used indicator—the share of retail cigarette prices that are accounted for by taxes—captures one aspect of cigarette taxes, but countries can have high tax shares and still see low cigarette prices and increasing cigarette affordability. Moreover, the tax share will not pick up the strengths and weaknesses of countries' tax structures, with weak tax structures creating greater variability in cigarette prices that allow smokers to trade down to cheaper brands when taxes rise, limiting the health and revenue benefits of higher taxes.

The four-component measure developed in this report has several limitations. It does not include a measure of the effectiveness of tax administration, which is critical for minimizing tax avoidance and evasion. As a result, the Scorecard may overstate the strength of tax systems in some countries with high taxes and prices, falling affordability, and good tax structures. For example, while Ecuador (4.38) and Montenegro (3.88) are among the highest scoring countries, both have significant problems with cigarette tax evasion, which limits the effectiveness of their cigarette taxes in reducing smoking and raising revenues. To some extent, the tax structure component will pick up aspects of tax administration, given that simple uniform specific excise taxes are easier to administer and create fewer opportunities for tax avoidance and evasion, but this component will miss other key aspects of tax administration.

A second limitation is the focus on cigarette taxation, given the lack of comprehensive data on the taxation of other tobacco products. This is particularly true for countries in which consumption of other tobacco products—including bidis, smokeless tobacco, and water pipe tobacco—is high; nor does it account for newer products like e-cigarettes and heated tobacco products. To the extent that taxes and prices on these products are low, relative to cigarette taxes and prices, there will be opportunities for substitution to the relatively cheaper products, reducing the health and revenue benefits of effective cigarette taxes.

Several of the components that comprise the overall score—including cigarette price, changes in affordability, and tax shares—are limited to the most-sold brand of cigarettes in each country. As a result, they do not reflect the variability in cigarette prices and the opportunities for smokers to switch to cheaper brands as cigarette taxes and prices rise. Again, this is partially, but not fully, captured by the tax structure component, given that the tax structures that score highest are those that reduce variability in prices across cigarette brands.

Additionally, some components are highly dependent on cigarette companies' pricing strategies. To the extent that cigarette companies raise prices by more than the amount of a tax increase, the tax share component may not fully reflect the aggressive tax increases implemented in countries like Australia and New Zealand. Alternatively, some countries may score well on the affordability component despite modest cigarette tax increases, if cigarette companies are increasing prices by much more than taxes are rising. Likewise, if industry prices are very low, tax shares can be very high but retail prices can be low and cigarettes can be highly affordable. To some extent, the multiple components of the overall score address some of these limitations, albeit imperfectly.

Finally, the thresholds used in determining the scores for the individual components are to a large extent arbitrary. That said, these thresholds are in part based on relevant recommendations and empirical evidence, as well as on the distribution of the data for each component. While changes in the thresholds would change the component-specific and overall scores, changes would have less impact on the relative scores.

Despite these limitations, this Scorecard provides the most comprehensive assessment of cigarette tax systems to date. As more comprehensive, consistently collected data on tax administration, other tobacco product taxes, and other factors become available, the Scorecard will be refined and improved. In the meantime, this Scorecard clearly demonstrates that there is considerable room for improving cigarette tax systems in the vast majority of countries and provides some indication of the weaknesses in current systems that could be readily addressed by tobacco tax policy makers. Policy makers could quickly increase their scores by sharply increasing cigarette taxes and moving towards simpler tax structures that rely on specific excise taxes. Making these improvements would save countless lives while generating much needed government revenue.

### Historic note:

This first version of the Tobacconomics Cigarette Tax Scorecard comes during the COVID-19 pandemic. As countries seek to improve the health of their populations and to respond to the crushing impact of the pandemic on their health systems, increasing and reforming tobacco tax systems is critical. Higher and better-designed tobacco taxes will reduce tobacco use and improve health, reducing the burden of tobacco-attributable diseases on health systems. At the same time, these taxes will generate substantial new revenues that can be used to rebuild health systems and support economic recovery. The low scores for the vast majority of countries in this Scorecard demonstrate that there is considerable room for countries to improve their cigarette tax systems to save countless lives and bring in much needed government revenue.

# *References*


Goodchild, M., Perucic, A. M., & Paul, J. (2020). *Tobacco taxation as a strategy to achieve global targets for smoking prevalence* [Unpublished manuscript]. Geneva, CH: World Health Organization.

Jha, P., & Chaloupka, F. J. (1999). *Curbing the epidemic: governments and the economics of tobacco control.* Washington, DC: World Bank Group. http://documents1.worldbank.org/curated/en/914041468176678949/pdf/multi-page.pdf

U.S. National Cancer Institute & World Health Organization NCI & WHO. (2018). *The economics of tobacco and tobacco control.* National Cancer Institute Tobacco Control Monograph 21. NIH Publication No. 16-CA-8029A. Bethesda, MD: U.S. Department of Health and Human Services, National Institutes of Health, National Cancer Institute; and Geneva, CH: World Health Organization. https://cancercontrol.cancer.gov/sites/default/files/2020-06/m21_complete.pdf

World Bank. (2017). *Tobacco tax reform at the crossroads of health and development: A multisectoral perspective.* https://openknowledge.worldbank.org/handle/10986/28494

World Health Organization. (2003). *WHO framework convention on tobacco control.* https://apps.who.int/iris/bitstream/handle/10665/42811/9241591013.pdf

World Health Organization. (2010). *Technical manual on tobacco tax administration.* https://apps.who.int/iris/bitstream/handle/10665/44316/9789241563994_eng.pdf

World Health Organization. (2014). *Guidelines for implementation of Article 6 of WHO FCTC.* http://www.who.int/fctc/treaty_instruments/Guidelines_article_6.pdf

World Health Organization. (2015). *WHO report on the global tobacco epidemic, 2015.* https://apps.who.int/iris/bitstream/handle/10665/178574/9789240694606_eng.pdf

World Health Organization. (2017). *WHO report on the global tobacco epidemic, 2017*. https://www.who.int/tobacco/global_report/2017/en

World Health Organization. (2019). *WHO report on the global tobacco epidemic, 2019*. https://www.who.int/publications/i/item/9789241516204

# Appendices

**Appendix Table 1** Overall cigarette tax scores, 2018

| Country | Overall score (2018) | Country | Overall score (2018) |
|---|---|---|---|
| Australia | 4.63 | Netherlands | 3.13 |
| New Zealand | 4.63 | Romania | 3.13 |
| Ecuador | 4.38 | Samoa | 3.13 |
| United Kingdom of Great Britain and Northern Ireland | 4.38 | Seychelles | 3.13 |
| | | Ukraine | 3.13 |
| Montenegro | 3.88 | Italy | 3.00 |
| Palau | 3.88 | Kazakhstan | 3.00 |
| Bahrain | 3.75 | Turkmenistan | 3.00 |
| Canada | 3.75 | Cyprus | 2.88 |
| Philippines | 3.75 | Estonia | 2.88 |
| Saudi Arabia | 3.75 | Germany | 2.88 |
| Bosnia and Herzegovina | 3.63 | Hungary | 2.88 |
| Chile | 3.63 | Kyrgyzstan | 2.88 |
| France | 3.63 | Latvia | 2.88 |
| Greece | 3.63 | Malaysia | 2.88 |
| Israel | 3.63 | Portugal | 2.88 |
| Norway | 3.63 | Slovakia | 2.88 |
| Peru | 3.63 | Slovenia | 2.88 |
| Serbia | 3.63 | Turkey | 2.88 |
| Tonga | 3.63 | Czechia | 2.75 |
| Argentina | 3.50 | Egypt | 2.75 |
| United Arab Emirates | 3.50 | Fiji | 2.75 |
| Finland | 3.38 | Poland | 2.75 |
| Jordan | 3.38 | Sweden | 2.75 |
| Lithuania | 3.38 | North Macedonia | 2.63 |
| Russian Federation | 3.38 | Spain | 2.63 |
| Sri Lanka | 3.38 | Zimbabwe | 2.63 |
| Suriname | 3.38 | Algeria | 2.50 |
| Belgium | 3.25 | Austria | 2.50 |
| Ireland | 3.25 | Croatia | 2.50 |
| Mauritius | 3.25 | Iceland | 2.50 |
| Singapore | 3.25 | Republic of Korea | 2.50 |
| Trinidad and Tobago | 3.25 | South Africa | 2.50 |
| Bulgaria | 3.13 | Switzerland | 2.50 |
| Gambia | 3.13 | Uruguay | 2.50 |
| Jamaica | 3.13 | Bangladesh | 2.38 |
| Malta | 3.13 | Botswana | 2.38 |

| Country | Overall score (2018) | Country | Overall score (2018) |
|---|---|---|---|
| Colombia | 2.38 | Belize | 1.50 |
| Denmark | 2.38 | Japan | 1.50 |
| Dominican Republic | 2.38 | Nauru | 1.50 |
| Luxembourg | 2.38 | Saint Vincent and the Grenadines | 1.50 |
| Morocco | 2.38 | Sierra Leone | 1.50 |
| Tajikistan | 2.38 | Burundi | 1.38 |
| Albania | 2.25 | Georgia | 1.38 |
| Chad | 2.25 | Guatemala | 1.38 |
| Eswatini | 2.25 | China | 1.25 |
| Barbados | 2.13 | Dominica | 1.25 |
| Brazil | 2.13 | Guyana | 1.25 |
| Costa Rica | 2.13 | Maldives | 1.25 |
| Lesotho | 2.13 | Sao Tome and Principe | 1.25 |
| Madagascar | 2.13 | Comoros | 1.13 |
| Mexico | 2.13 | Congo | 1.13 |
| Vanuatu | 2.13 | Grenada | 1.13 |
| Namibia | 2.00 | Republic of Moldova | 1.13 |
| Sudan | 2.00 | Zambia | 1.13 |
| United States of America | 2.00 | Equatorial Guinea | 1.00 |
| Belarus | 1.88 | Gabon | 1.00 |
| El Salvador | 1.88 | Oman | 1.00 |
| India | 1.88 | Saint Kitts and Nevis | 1.00 |
| Panama | 1.88 | Tuvalu | 1.00 |
| Saint Lucia | 1.88 | Armenia | 0.88 |
| Honduras | 1.75 | Central African Republic | 0.88 |
| Kiribati | 1.75 | Kenya | 0.88 |
| Nepal | 1.75 | Lebanon | 0.88 |
| Papua New Guinea | 1.75 | Pakistan | 0.88 |
| Thailand | 1.75 | Qatar | 0.88 |
| Timor-Leste | 1.75 | Uzbekistan | 0.88 |
| Tunisia | 1.75 | Viet Nam | 0.88 |
| Burkina Faso | 1.63 | Benin | 0.75 |
| Indonesia | 1.63 | Cabo Verde | 0.75 |
| Mongolia | 1.63 | Cameroon | 0.75 |
| Nicaragua | 1.63 | Côte d'Ivoire | 0.75 |
| Rwanda | 1.63 | Ghana | 0.75 |
| Senegal | 1.63 | Guinea-Bissau | 0.75 |

## Appendix Table 1 Overall cigarette tax scores, 2018

| Country | Overall score (2018) | Country | Overall score (2018) |
|---|---|---|---|
| Kuwait | 0.75 | Afghanistan | 0.25 |
| Mauritania | 0.75 | Libya | 0.25 |
| Micronesia (Federated States of) | 0.75 | Iraq | 0.00 |
| Niger | 0.75 | Andorra | . |
| Nigeria | 0.75 | Bahamas | . |
| Solomon Islands | 0.75 | Bhutan | . |
| Togo | 0.75 | Brunei Darussalam | . |
| Uganda | 0.75 | Cook Islands | . |
| United Republic of Tanzania | 0.75 | Cuba | . |
| Azerbaijan | 0.63 | Democratic People's Republic of Korea | . |
| Bolivia (Plurinational State of) | 0.63 | Djibouti | . |
| Democratic Republic of the Congo | 0.63 | Eritrea | . |
| Myanmar | 0.63 | Guinea | . |
| Angola | 0.50 | Haiti | . |
| Antigua and Barbuda | 0.50 | Malawi | . |
| Cambodia | 0.50 | Monaco | . |
| Ethiopia | 0.50 | Niue | . |
| Iran (Islamic Republic of) | 0.50 | San Marino | . |
| Lao People's Democratic Republic | 0.50 | Somalia | . |
| Liberia | 0.50 | South Sudan | . |
| Mali | 0.50 | Syrian Arab Republic | . |
| Marshall Islands | 0.50 | Venezuela (Bolivarian Republic of) | . |
| Mozambique | 0.50 | West Bank and Gaza Strip | . |
| Paraguay | 0.50 | Yemen | . |

## Appendix Table 2-A Overall and component cigarette tax scores, 2018

| Country | Absolute Price (2018) | Affordability Change (2018) | Tax Share (2018) | Tax Structure (2018) | OVERALL (2018) |
|---|---|---|---|---|---|
| Afghanistan | 0 | 1 | 0 | 0 | 0.25 |
| Albania | 2 | 0 | 3 | 4 | 2.25 |
| Algeria | 4 | 5 | 0 | 1 | 2.50 |
| Andorra | . | 0 | 5 | 4 | . |
| Angola | 1 | 1 | 0 | 0 | 0.50 |
| Antigua and Barbuda | 2 | 0 | 0 | 0 | 0.50 |
| Argentina | 2 | 5 | 5 | 2 | 3.50 |
| Armenia | 1 | 0* | 0.5 | 2 | 0.88 |
| Australia | 5 | 4 | 4.5 | 5 | 4.63 |
| Austria | 3 | 0 | 4 | 3 | 2.50 |
| Azerbaijan | 1 | 0 | 0.5 | 1 | 0.63 |
| Bahamas | . | 5 | . | . | . |
| Bahrain | 5 | 5 | 3 | 2 | 3.78 |
| Bangladesh | 1 | 4 | 3.5 | 1 | 2.38 |
| Barbados | 3 | 0 | 1.5 | 4 | 2.13 |
| Belarus | 0 | 5 | 1.5 | 1 | 1.88 |
| Belgium | 4 | 2 | 4 | 3 | 3.25 |
| Belize | 1 | 0* | 1 | 4 | 1.50 |
| Benin | 1 | 0* | 0 | 2 | 0.75 |
| Bhutan | . | . | . | . | . |
| Bolivia (Plurinational State of) | 1 | 0 | 0.5 | 1 | 0.63 |
| Bosnia and Herzegovina | 3 | 4 | 4.5 | 3 | 3.63 |
| Botswana | 4 | 0 | 1.5 | 4 | 2.38 |
| Brazil | 1 | 0 | 3.5 | 4 | 2.13 |
| Brunei Darussalam | . | . | . | . | . |
| Bulgaria | 4 | 0* | 4.5 | 4 | 3.13 |
| Burkina Faso | 1 | 3 | 0.5 | 2 | 1.63 |
| Burundi | 1 | 0 | 0.5 | 4 | 1.38 |
| Cabo Verde | 1 | 0* | 0 | 2 | 0.75 |
| Cambodia | 0 | 0* | 0 | 2 | 0.50 |
| Cameroon | 1 | 0* | 0 | 2 | 0.75 |
| Canada | 4 | 3 | 3 | 5 | 3.75 |
| Central African Republic | 0 | 0 | 0.5 | 3 | 0.88 |
| Chad | 2 | 5 | 0 | 2 | 2.25 |
| Chile | 3 | 3 | 4.5 | 4 | 3.63 |
| China | 2 | 0* | 2 | 1 | 1.25 |
| Colombia | 1 | 0 | 4.5 | 4 | 2.38 |
| Comoros | 1 | 1 | 0.5 | 2 | 1.13 |
| Congo | 1 | 0 | 0.5 | 3 | 1.13 |
| Cook Islands | . | 0 | 4 | 4 | . |

## Appendix Table 2-A Overall and component cigarette tax scores, 2018

| Country | 2018 | | | | |
|---|---|---|---|---|---|
| | Absolute Price | Affordability Change | Tax Share | Tax Structure | OVERALL |
| Costa Rica | 2 | 0 | 2.5 | 4 | 2.13 |
| Côte d'Ivoire | 1 | 0 | 0 | 2 | 0.75 |
| Croatia | 3 | 0* | 4 | 3 | 2.50 |
| Cuba | . | 0 | 4.5 | 2 | . |
| Cyprus | 3 | 2 | 3.5 | 3 | 2.88 |
| Czechia | 3 | 0* | 4 | 4 | 2.75 |
| Democratic People's Republic of Korea | . | . | 0 | 0 | . |
| Democratic Republic of the Congo | 0 | 0 | 0.5 | 2 | 0.63 |
| Denmark | 2 | 0 | 3.5 | 4 | 2.38 |
| Djibouti | . | 0* | . | . | . |
| Dominica | 1 | 0 | 0 | 4 | 1.25 |
| Dominican Republic | 4 | 0 | 1.5 | 4 | 2.38 |
| Ecuador | 5 | 5 | 3.5 | 4 | 4.38 |
| Egypt | 2 | 3 | 5 | 1 | 2.75 |
| El Salvador | 3 | 0 | 1.5 | 3 | 1.88 |
| Equatorial Guinea | 1 | 1 | 0 | 2 | 1.00 |
| Eritrea | . | 0 | 2.5 | 2 | . |
| Estonia | 3 | 0 | 4.5 | 4 | 2.88 |
| Eswatini | 3 | 0* | 2 | 4 | 2.25 |
| Ethiopia | 0 | 0 | 0 | 2 | 0.50 |
| Fiji | 5 | 4 | 1 | 1 | 2.75 |
| Finland | 3 | 3 | 4.5 | 3 | 3.38 |
| France | 5 | 2 | 4.5 | 3 | 3.63 |
| Gabon | 2 | 0 | 0 | 2 | 1.00 |
| Gambia | 2 | 5 | 1.5 | 4 | 3.13 |
| Georgia | 1 | 0 | 3.5 | 1 | 1.38 |
| Germany | 4 | 0 | 3.5 | 4 | 2.88 |
| Ghana | 1 | 0 | 0 | 2 | 0.75 |
| Greece | 3 | 3 | 4.5 | 4 | 3.63 |
| Grenada | 2 | 0 | 0.5 | 2 | 1.13 |
| Guatemala | 2 | 0 | 1.5 | 2 | 1.38 |
| Guinea | . | 0 | . | . | . |
| Guinea-Bissau | 1 | 0 | 0 | 2 | 0.75 |
| Guyana | 1 | 0 | 0 | 4 | 1.25 |
| Haiti | . | . | . | . | . |
| Honduras | 2 | 0 | 0 | 5 | 1.75 |
| Hungary | 4 | 0 | 3.5 | 4 | 2.88 |
| Iceland | 4 | 0 | 2 | 4 | 2.50 |
| India | 5 | 0 | 1.5 | 1 | 1.88 |

| | 2018 | | | | |
|---|---|---|---|---|---|
| Country | Absolute Price | Affordability Change | Tax Share | Tax Structure | OVERALL |
| Indonesia | 3 | 0 | 2.5 | 1 | 1.63 |
| Iran (Islamic Republic of) | 1 | 0 | 0 | 1 | 0.50 |
| Iraq | 0 | 0 | 0 | 0 | 0.00 |
| Ireland | 5 | 0* | 4 | 4 | 3.25 |
| Israel | 4 | 3 | 4.5 | 3 | 3.63 |
| Italy | 3 | 2 | 4 | 3 | 3.00 |
| Jamaica | 5 | 3 | 0.5 | 4 | 3.13 |
| Japan | 2 | 0* | 3 | 1 | 1.50 |
| Jordan | 3 | 5 | 4.5 | 1 | 3.38 |
| Kazakhstan | 1 | 5 | 2 | 4 | 3.00 |
| Kenya | 1 | 0* | 1.5 | 1 | 0.88 |
| Kiribati | 2 | 0 | 1 | 4 | 1.75 |
| Kuwait | 2 | 1 | 0 | 0 | 0.75 |
| Kyrgyzstan | 1 | 5 | 1.5 | 4 | 2.88 |
| Lao People's Democratic Republic | 1 | 0* | 0 | 1 | 0.50 |
| Latvia | 3 | 0 | 4.5 | 4 | 2.88 |
| Lebanon | 1 | 0 | 1.5 | 1 | 0.88 |
| Lesotho | 3 | 0* | 1.5 | 4 | 2.13 |
| Liberia | 0 | 0 | 0 | 2 | 0.50 |
| Libya | 1 | 0 | 0 | 0 | 0.25 |
| Lithuania | 4 | 2 | 3.5 | 4 | 3.38 |
| Luxembourg | 3 | 0 | 3.5 | 3 | 2.38 |
| Madagascar | 2 | 0 | 4.5 | 2 | 2.13 |
| Malawi | . | 0 | . | . | . |
| Malaysia | 5 | 0 | 2.5 | 4 | 2.88 |
| Maldives | 3 | 0 | 2 | 0 | 1.25 |
| Mali | 1 | 0 | 0 | 1 | 0.50 |
| Malta | 4 | 0* | 4.5 | 4 | 3.13 |
| Marshall Islands | 1 | 0 | 1 | 0 | 0.50 |
| Mauritania | 1 | 0 | 0 | 2 | 0.75 |
| Mauritius | 4 | 0 | 5 | 4 | 3.25 |
| Mexico | 2 | 0 | 3.5 | 3 | 2.13 |
| Micronesia (Federated States of) | 1 | 1 | 1 | 0 | 0.75 |
| Monaco | . | . | . | . | . |
| Mongolia | 1 | 0 | 1.5 | 4 | 1.63 |
| Montenegro | 3 | 5 | 4.5 | 3 | 3.88 |
| Morocco | 2 | 0 | 3.5 | 4 | 2.38 |
| Mozambique | 1 | 0 | 0 | 1 | 0.50 |
| Myanmar | 1 | 0* | 0.5 | 1 | 0.63 |
| Namibia | 3 | 0 | 1 | 4 | 2.00 |

## Appendix Table 2-A Overall and component cigarette tax scores, 2018

| Country | Absolute Price (2018) | Affordability Change (2018) | Tax Share (2018) | Tax Structure (2018) | OVERALL (2018) |
|---|---|---|---|---|---|
| Nauru | 5 | 0 | 1 | 0 | 1.50 |
| Nepal | 3 | 3 | 0 | 1 | 1.75 |
| Netherlands | 4 | 0 | 3.5 | 5 | 3.13 |
| New Zealand | 5 | 4 | 4.5 | 5 | 4.63 |
| Nicaragua | 1 | 0 | 0.5 | 5 | 1.63 |
| Niger | 1 | 0 | 0 | 2 | 0.75 |
| Nigeria | 0 | 0* | 0 | 3 | 0.75 |
| Niue | . | . | 2.5 | 0 | . |
| North Macedonia | 1 | 0 | 4.5 | 5 | 2.63 |
| Norway | 5 | 3 | 2.5 | 4 | 3.63 |
| Oman | 3 | 1 | 0 | 0 | 1.00 |
| Pakistan | 0 | 0 | 2.5 | 1 | 0.88 |
| Palau | 3 | 4 | 4.5 | 4 | 3.88 |
| Panama | 3 | 0* | 2.5 | 2 | 1.88 |
| Papua New Guinea | 4 | 0 | 2 | 1 | 1.75 |
| Paraguay | 0 | 0 | 0 | 2 | 0.50 |
| Peru | 4 | 5 | 1.5 | 4 | 3.63 |
| Philippines | 1 | 5 | 4 | 5 | 3.75 |
| Poland | 4 | 0 | 4 | 3 | 2.75 |
| Portugal | 4 | 0 | 3.5 | 4 | 2.88 |
| Qatar | 2 | 1 | 0.5 | 0 | 0.88 |
| Republic of Korea | 2 | 0 | 4 | 4 | 2.50 |
| Republic of Moldova | 1 | 0 | 2.5 | 1 | 1.13 |
| Romania | 4 | 0* | 3.5 | 5 | 3.13 |
| Russian Federation | 2 | 5 | 2.5 | 4 | 3.38 |
| Rwanda | 1 | 0* | 2.5 | 3 | 1.63 |
| Saint Kitts and Nevis | 2 | 0* | 0 | 2 | 1.00 |
| Saint Lucia | 2 | 0 | 1.5 | 4 | 1.88 |
| Saint Vincent and the Grenadines | 2 | 0 | 0 | 4 | 1.50 |
| Samoa | 3 | 4 | 1.5 | 4 | 3.13 |
| San Marino | . | 1 | . | . | . |
| Sao Tome and Principe | 0 | 0 | 1 | 4 | 1.25 |
| Saudi Arabia | 5 | 5 | 3 | 2 | 3.75 |
| Senegal | 1 | 3 | 0.5 | 2 | 1.63 |
| Serbia | 2 | 5 | 4.5 | 3 | 3.63 |
| Seychelles | 5 | 0 | 3.5 | 4 | 3.13 |
| Sierra Leone | 1 | 3 | 0 | 2 | 1.50 |
| Singapore | 5 | 0 | 4 | 4 | 3.25 |
| Slovakia | 3 | 0 | 4.5 | 4 | 2.88 |

| | | 2018 | | | |
|---|---|---|---|---|---|
| **Country** | **Absolute Price** | **Affordability Change** | **Tax Share** | **Tax Structure** | **OVERALL** |
| Slovenia | 3 | 0 | 4.5 | 4 | 2.88 |
| Solomon Islands | 2 | 0 | 0 | 1 | 0.75 |
| Somalia | . | . | 0 | 0 | . |
| South Africa | 3 | 0 | 2 | 5 | 2.50 |
| South Sudan | . | . | . | . | . |
| Spain | 3 | 0 | 4.5 | 3 | 2.63 |
| Sri Lanka | 5 | 4 | 3.5 | 1 | 3.38 |
| Sudan | 2 | 0 | 4 | 2 | 2.00 |
| Suriname | 3 | 5 | 1.5 | 4 | 3.38 |
| Sweden | 3 | 0 | 3 | 5 | 2.75 |
| Switzerland | 3 | 0 | 3 | 4 | 2.50 |
| Syrian Arab Republic | . | . | 0.5 | 2 | . |
| Tajikistan | 1 | 4 | 0.5 | 4 | 2.38 |
| Thailand | 2 | 0 | 4 | 1 | 1.75 |
| Timor-Leste | 2 | 1 | 0 | 4 | 1.75 |
| Togo | 1 | 0 | 0 | 2 | 0.75 |
| Tonga | 5 | 5 | 3.5 | 1 | 3.63 |
| Trinidad and Tobago | 4 | 5 | 0 | 4 | 3.25 |
| Tunisia | 1 | 0* | 3 | 3 | 1.75 |
| Turkey | 4 | 0* | 4.5 | 3 | 2.88 |
| Turkmenistan | 5 | 5 | 0 | 2 | 3.00 |
| Tuvalu | 2 | 0 | 0 | 2 | 1.00 |
| Uganda | 1 | 0 | 1 | 1 | 0.75 |
| Ukraine | 1 | 4 | 3.5 | 4 | 3.13 |
| United Arab Emirates | 4 | 5 | 3 | 2 | 3.50 |
| United Kingdom of Great Britain and Northern Ireland | 5 | 3 | 4.5 | 5 | 4.38 |
| United Republic of Tanzania | 2 | 0 | 0 | 1 | 0.75 |
| United States of America | 3 | 0* | 1 | 4 | 2.00 |
| Uruguay | 3 | 0 | 3 | 4 | 2.50 |
| Uzbekistan | 2 | 0 | 0.5 | 1 | 0.88 |
| Vanuatu | 2 | 0* | 2.5 | 4 | 2.13 |
| Venezuela (Bolivarian Republic of) | . | 1 | 4 | 2 | . |
| Viet Nam | 1 | 0 | 0.5 | 2 | 0.88 |
| West Bank and Gaza Strip | . | 0 | 4.5 | 4 | . |
| Yemen | . | 4 | 1 | 4 | . |
| Zambia | 2 | 0 | 0.5 | 2 | 1.13 |
| Zimbabwe | 1 | 5 | 0.5 | 4 | 2.63 |
| Global Average | 2.35 | 1.18 | 2.06 | 2.69 | 2.07 |

Note: Overall scores are rounded to two decimal places. Countries in each column are listed in order of their scores, from lowest to highest, and alphabetically when scores are identical.

## Appendix Table 2-B Overall and component cigarette tax scores, 2016

| Country | Absolute Price | Affordability Change | Tax Share | Tax Structure | OVERALL |
|---|---|---|---|---|---|
| | | | 2016 | | |
| Afghanistan | 0 | 1 | 0 | 0 | 0.25 |
| Albania | 2 | 3 | 3 | 4 | 3.00 |
| Algeria | 3 | 5 | 0.5 | 1 | 2.38 |
| Andorra | . | 0 | 4.5 | 1 | . |
| Angola | . | 0 | . | . | . |
| Antigua and Barbuda | 2 | 0 | 0 | 0 | 0.50 |
| Argentina | 1 | 0 | 5 | 2 | 2.00 |
| Armenia | 1 | 0* | 0.5 | 1 | 0.63 |
| Australia | 5 | 4 | 3 | 5 | 4.25 |
| Austria | 2 | 2 | 4 | 3 | 2.75 |
| Azerbaijan | 3 | 5 | 0 | 4 | 3.00 |
| Bahamas | 4 | 5 | 1 | 4 | 3.50 |
| Bahrain | 3 | 1 | 0 | 0 | 1.00 |
| Bangladesh | 0 | 0 | 3.5 | 1 | 1.13 |
| Barbados | 3 | 3 | 0.5 | 4 | 2.63 |
| Belarus | 0 | 0 | 1.5 | 1 | 0.63 |
| Belgium | 3 | 3 | 4 | 3 | 3.25 |
| Belize | 2 | 0* | 0.5 | 4 | 1.63 |
| Benin | 1 | 0* | 0 | 2 | 0.75 |
| Bhutan | . | . | . | . | . |
| Bolivia (Plurinational State of) | 1 | 1 | 0.5 | 1 | 0.88 |
| Bosnia and Herzegovina | 3 | 5 | 4.5 | 3 | 3.88 |
| Botswana | 3 | 0 | 1.5 | 4 | 2.13 |
| Brazil | 1 | 3 | 2.5 | 5 | 2.88 |
| Brunei Darussalam | . | 0 | . | . | . |
| Bulgaria | 3 | 0* | 4.5 | 3 | 2.63 |
| Burkina Faso | 1 | 0 | 0 | 1 | 0.50 |
| Burundi | 1 | 0 | 0.5 | 4 | 1.38 |
| Cabo Verde | 1 | 0* | 0 | 2 | 0.75 |
| Cambodia | 0 | 0 | 0 | 2 | 0.50 |
| Cameroon | 1 | 0* | 0 | 2 | 0.75 |
| Canada | 4 | 0 | 3.5 | 5 | 3.13 |
| Central African Republic | . | 0 | . | . | . |
| Chad | 1 | 5 | 0 | 2 | 2.00 |
| Chile | 2 | 3 | 5 | 4 | 3.50 |
| China | 1 | 0 | 2 | 1 | 1.00 |
| Colombia | 1 | 0* | 1.5 | 4 | 1.63 |
| Comoros | 1 | 0 | 0.5 | 2 | 0.88 |
| Congo | 1 | 5 | 0.5 | 3 | 2.38 |
| Cook Islands | . | 5 | 4 | 4 | . |

| | 2016 | | | | |
|---|---|---|---|---|---|
| Country | Absolute Price | Affordability Change | Tax Share | Tax Structure | OVERALL |
| Costa Rica | 2 | 0 | 2.5 | 4 | 2.13 |
| Côte d'Ivoire | 1 | 3 | 0 | 2 | 1.50 |
| Croatia | 3 | 0* | 4 | 3 | 2.50 |
| Cuba | . | 1 | 4.5 | 2 | . |
| Cyprus | 3 | 2 | 4.5 | 3 | 3.13 |
| Czechia | 2 | 0* | 4.5 | 4 | 2.63 |
| Democratic People's Republic of Korea | . | . | 0 | 0 | . |
| Democratic Republic of the Congo | 0 | 0 | 0 | 1 | 0.25 |
| Denmark | 2 | 2 | 3.5 | 4 | 2.88 |
| Djibouti | 0 | 0* | 0 | 2 | 0.50 |
| Dominica | 1 | 0 | 0 | 4 | 1.25 |
| Dominican Republic | 3 | 0* | 2.5 | 4 | 2.38 |
| Ecuador | 4 | 5 | 4 | 5 | 4.50 |
| Egypt | 2 | 3 | 4.5 | 1 | 2.63 |
| El Salvador | 1 | 0* | 2 | 4 | 1.75 |
| Equatorial Guinea | 0 | 0 | 0 | 2 | 0.50 |
| Eritrea | . | 1 | 2.5 | 2 | . |
| Estonia | 3 | 3 | 4.5 | 4 | 3.63 |
| Eswatini | 3 | 0* | 1.5 | 5 | 2.38 |
| Ethiopia | 0 | 0 | 0 | 2 | 0.50 |
| Fiji | 4 | 0 | 1 | 1 | 1.50 |
| Finland | 3 | 3 | 4.5 | 3 | 3.38 |
| France | 4 | 3 | 4.5 | 3 | 3.63 |
| Gabon | 1 | 0 | 0 | 2 | 0.75 |
| Gambia | 1 | 5 | 1.5 | 4 | 2.88 |
| Georgia | 1 | 0* | 3.5 | 1 | 1.38 |
| Germany | 3 | 0 | 3.5 | 4 | 2.63 |
| Ghana | 1 | 0 | 0 | 2 | 0.75 |
| Greece | 3 | 4 | 4.5 | 4 | 3.88 |
| Grenada | 2 | 0* | 1.5 | 2 | 1.38 |
| Guatemala | 2 | 0* | 1.5 | 2 | 1.38 |
| Guinea | 0 | 0 | 0 | 2 | 0.50 |
| Guinea-Bissau | 0 | 0 | 0 | 2 | 0.50 |
| Guyana | 1 | 0* | 0 | 2 | 0.75 |
| Haiti | 1 | . | 0 | 2 | . |
| Honduras | 1 | 0 | 0 | 5 | 1.50 |
| Hungary | 4 | 4 | 3.5 | 4 | 3.88 |
| Iceland | 4 | 0 | 2 | 4 | 2.50 |
| India | 4 | 3 | 1.5 | 1 | 2.38 |

## Appendix Table 2-B Overall and component cigarette tax scores, 2016

| Country | 2016 | | | | |
|---|---|---|---|---|---|
| | Absolute Price | Affordability Change | Tax Share | Tax Structure | OVERALL |
| Indonesia | 2 | 0 | 2.5 | 1 | 1.38 |
| Iran (Islamic Republic of) | 1 | 0 | 0 | 4 | 1.25 |
| Iraq | 0 | 0 | 0 | 0 | 0.00 |
| Ireland | 5 | 0 | 4 | 4 | 3.25 |
| Israel | 4 | 4 | 4.5 | 3 | 3.88 |
| Italy | 3 | 0 | 4 | 3 | 2.50 |
| Jamaica | 5 | 2 | 0.5 | 4 | 2.88 |
| Japan | 2 | 0 | 3 | 1 | 1.50 |
| Jordan | 1 | 3 | 4.5 | 1 | 2.38 |
| Kazakhstan | 1 | 4 | 1.5 | 4 | 2.63 |
| Kenya | 1 | 0* | 1.5 | 4 | 1.63 |
| Kiribati | 2 | 0 | 1 | 4 | 1.75 |
| Kuwait | 2 | 1 | 0 | 0 | 0.75 |
| Kyrgyzstan | 1 | 0 | 0.5 | 1 | 0.63 |
| Lao People's Democratic Republic | 1 | 0 | 0 | 4 | 1.25 |
| Latvia | 2 | 0 | 4.5 | 4 | 2.63 |
| Lebanon | 1 | 0 | 1 | 2 | 1.00 |
| Lesotho | . | 0* | . | . | . |
| Liberia | 0 | 0* | 0 | 2 | 0.50 |
| Libya | 1 | 1 | 0 | 0 | 0.50 |
| Lithuania | 3 | 0 | 4 | 4 | 2.75 |
| Luxembourg | 2 | 3 | 3.5 | 3 | 2.88 |
| Madagascar | 2 | 4 | 4.5 | 2 | 3.13 |
| Malawi | . | 0 | . | . | . |
| Malaysia | 5 | 0 | 2 | 4 | 2.75 |
| Maldives | 2 | 0 | 1 | 0 | 0.75 |
| Mali | 1 | 0 | 0 | 1 | 0.50 |
| Malta | 4 | 0* | 4.5 | 4 | 3.13 |
| Marshall Islands | 1 | 0 | 1 | 0 | 0.50 |
| Mauritania | 1 | 0 | 0 | 2 | 0.75 |
| Mauritius | 3 | 0 | 3.5 | 4 | 2.63 |
| Mexico | 2 | 0 | 3.5 | 3 | 2.13 |
| Micronesia (Federated States of) | 1 | 1 | 1.5 | 0 | 0.88 |
| Monaco | . | . | . | . | . |
| Mongolia | 1 | 0 | 2 | 4 | 1.75 |
| Montenegro | 2 | 5 | 3.5 | 3 | 3.38 |
| Morocco | 2 | 0 | 3.5 | 1 | 1.63 |
| Mozambique | 0 | 0 | 0 | 1 | 0.25 |
| Myanmar | 1 | 0* | 1 | 1 | 0.75 |

| 2016 | | | | | |
|---|---|---|---|---|---|
| **Country** | **Absolute Price** | **Affordability Change** | **Tax Share** | **Tax Structure** | **OVERALL** |
| Namibia | 3 | 0 | 1 | 4 | 2.00 |
| Nauru | 5 | 0 | 1 | 0 | 1.50 |
| Nepal | 2 | 0 | 0 | 1 | 0.75 |
| Netherlands | 3 | 3 | 3.5 | 5 | 3.63 |
| New Zealand | 5 | 5 | 4 | 5 | 4.75 |
| Nicaragua | 2 | 0 | 0.5 | 4 | 1.63 |
| Niger | 1 | 0 | 0.5 | 2 | 0.88 |
| Nigeria | 1 | 0* | 0 | 2 | 0.75 |
| Niue | . | . | 2.5 | 0 | . |
| North Macedonia | 1 | 0* | 3.5 | 5 | 2.38 |
| Norway | 5 | 3 | 3 | 4 | 3.75 |
| Oman | 3 | 1 | 0 | 0 | 1.00 |
| Pakistan | 1 | 4 | 3 | 1 | 2.25 |
| Palau | 2 | 0 | 4.5 | 4 | 2.63 |
| Panama | 3 | 0* | 2.5 | 2 | 1.88 |
| Papua New Guinea | 5 | 0 | 0.5 | 1 | 1.63 |
| Paraguay | 0 | 0 | 0 | 2 | 0.50 |
| Peru | 3 | 4 | 1.5 | 4 | 3.13 |
| Philippines | 1 | 5 | 3 | 1 | 2.50 |
| Poland | 4 | 4 | 4 | 3 | 3.75 |
| Portugal | 3 | 0 | 3.5 | 4 | 2.63 |
| Qatar | 2 | 1 | 0 | 0 | 0.75 |
| Republic of Korea | 2 | 0 | 4 | 4 | 2.50 |
| Republic of Moldova | 1 | 0 | 2.5 | 1 | 1.13 |
| Romania | 4 | 0 | 3.5 | 4 | 2.88 |
| Russian Federation | 2 | 5 | 1.5 | 4 | 3.13 |
| Rwanda | 1 | 0* | 2.5 | 3 | 1.63 |
| Saint Kitts and Nevis | 2 | 0 | 0 | 2 | 1.00 |
| Saint Lucia | 2 | 0 | 1.5 | 4 | 1.88 |
| Saint Vincent and the Grenadines | 1 | 0 | 0 | 4 | 1.25 |
| Samoa | 2 | 3 | 1.5 | 4 | 2.63 |
| San Marino | 3 | 1 | 4.5 | . | . |
| Sao Tome and Principe | 0 | 0* | 0 | 2 | 0.50 |
| Saudi Arabia | 3 | 1 | 0 | 0 | 1.00 |
| Senegal | 1 | 0 | 0.5 | 2 | 0.88 |
| Serbia | 2 | 5 | 4.5 | 3 | 3.63 |
| Seychelles | 5 | 0 | 3.5 | 4 | 3.13 |
| Sierra Leone | 0 | 0 | 0 | 0 | 0.00 |
| Singapore | 5 | 0 | 3.5 | 4 | 3.13 |

## Appendix Table 2-B Overall and component cigarette tax scores, 2016

| Country | 2016 Absolute Price | Affordability Change | Tax Share | Tax Structure | OVERALL |
|---|---|---|---|---|---|
| Slovakia | 3 | 2 | 4.5 | 4 | 3.38 |
| Slovenia | 2 | 3 | 4.5 | 4 | 3.38 |
| Solomon Islands | 1 | 0 | 0 | 1 | 0.50 |
| Somalia | . | . | 0 | 0 | . |
| South Africa | 2 | 0 | 1.5 | 4 | 1.88 |
| South Sudan | . | . | 0 | 2 | . |
| Spain | 3 | 0 | 4.5 | 3 | 2.63 |
| Sri Lanka | 5 | 0 | 2.5 | 1 | 2.13 |
| Sudan | 2 | 0 | 4 | 2 | 2.00 |
| Suriname | 2 | 5 | 3 | 1 | 2.75 |
| Sweden | 3 | 2 | 3 | 4 | 3.00 |
| Switzerland | 3 | 2 | 3 | 4 | 3.00 |
| Syrian Arab Republic | . | . | 0.5 | 2 | . |
| Tajikistan | 1 | 0 | 0 | 1 | 0.50 |
| Thailand | 3 | 0 | 4 | 2 | 2.25 |
| Timor-Leste | 1 | 0 | 0 | 4 | 1.25 |
| Togo | 1 | 0 | 0 | 2 | 0.75 |
| Tonga | 3 | 3 | 4 | 1 | 2.75 |
| Trinidad and Tobago | 3 | 5 | 0 | 4 | 3.00 |
| Tunisia | 1 | 0 | 3 | 3 | 1.75 |
| Turkey | 3 | 0 | 4.5 | 3 | 2.63 |
| Turkmenistan | 5 | 1 | 0 | 2 | 2.00 |
| Tuvalu | 1 | 0* | 2 | 2 | 1.25 |
| Uganda | 1 | 0 | 1 | 1 | 0.75 |
| Ukraine | 1 | 2 | 4.5 | 4 | 2.88 |
| United Arab Emirates | 2 | 1 | 0 | 0 | 0.75 |
| United Kingdom of Great Britain and Northern Ireland | 5 | 2 | 4.5 | 4 | 3.88 |
| United Republic of Tanzania | 2 | 0* | 0 | 1 | 0.75 |
| United States of America | 3 | 0* | 1 | 4 | 2.00 |
| Uruguay | 2 | 0 | 3 | 4 | 2.25 |
| Uzbekistan | 1 | 0 | 0.5 | 1 | 0.63 |
| Vanuatu | 2 | 0 | 2 | 4 | 2.00 |
| Venezuela (Bolivarian Republic of) | . | 1 | 4 | 2 | . |
| Viet Nam | 1 | 0 | 0.5 | 2 | 0.88 |
| West Bank and Gaza Strip | . | 0 | 4.5 | 4 | . |
| Yemen | . | 5 | 1 | 1 | . |
| Zambia | 2 | 0 | 0.5 | 2 | 1.13 |
| Zimbabwe | 1 | 5 | 0.5 | 4 | 2.63 |
| Global Average | 2.05 | 1.14 | 1.94 | 2.55 | 1.93 |

Note: Overall scores are rounded to two decimal places. Countries in each column are listed in order of their scores, from lowest to highest, and alphabetically when scores are identical.

## Appendix Table 2-C Overall and component cigarette tax scores, 2014

| Country | 2014 | | | | |
|---|---|---|---|---|---|
| | Absolute Price | Affordability Change | Tax Share | Tax Structure | OVERALL |
| Afghanistan | 0 | 0 | 0 | 0 | 0.00 |
| Albania | 2 | 3 | 2.5 | 4 | 2.88 |
| Algeria | 2 | 0 | 0.5 | 1 | 0.88 |
| Andorra | . | 5 | 4 | 1 | . |
| Angola | 1 | 1 | 0 | 0 | 0.50 |
| Antigua and Barbuda | 2 | 1 | 0 | 0 | 0.75 |
| Argentina | 1 | 0 | 4 | 2 | 1.75 |
| Armenia | 1 | 0 | 0 | 1 | 0.50 |
| Australia | 5 | 4 | 2.5 | 5 | 4.13 |
| Austria | 2 | 3 | 3.5 | 3 | 2.88 |
| Azerbaijan | 2 | 1 | 0 | 1 | 1.00 |
| Bahamas | 3 | 5 | 1.5 | 4 | 3.38 |
| Bahrain | 2 | 1 | 0 | 0 | 0.75 |
| Bangladesh | 0 | 0 | 2.5 | 1 | 0.88 |
| Barbados | 3 | 3 | 0.5 | 4 | 2.63 |
| Belarus | 0 | 0 | 1.5 | 1 | 0.63 |
| Belgium | 3 | 3 | 4 | 3 | 3.25 |
| Belize | 2 | 0* | 0.5 | 0 | 0.63 |
| Benin | 1 | 0* | 0 | 2 | 0.75 |
| Bhutan | . | . | . | . | . |
| Bolivia (Plurinational State of) | 1 | 0 | 0.5 | 2 | 0.88 |
| Bosnia and Herzegovina | 2 | 5 | 4.5 | 3 | 3.63 |
| Botswana | 3 | 0 | 2.5 | 4 | 2.38 |
| Brazil | 1 | 4 | 1.5 | 1 | 1.88 |
| Brunei Darussalam | . | 5 | . | . | . |
| Bulgaria | 3 | 0 | 4.5 | 4 | 2.88 |
| Burkina Faso | 1 | 0* | 0 | 1 | 0.50 |
| Burundi | 1 | 0 | 0.5 | 4 | 1.38 |
| Cabo Verde | 1 | 0* | 0 | 2 | 0.75 |
| Cambodia | 0 | 0 | 0 | 2 | 0.50 |
| Cameroon | 1 | 0* | 0 | . | . |
| Canada | 3 | 0 | 4 | 5 | 3.00 |
| Central African Republic | 0 | 1 | 0 | 2 | 0.75 |
| Chad | 1 | 0 | 0 | 2 | 0.75 |
| Chile | 2 | 0 | 4.5 | 3 | 2.38 |
| China | 1 | 0 | 1.5 | 1 | 0.88 |
| Colombia | 0 | 0* | 1.5 | 4 | 1.38 |
| Comoros | 1 | 0* | 1.5 | 2 | 1.13 |
| Congo | 1 | 0 | 0.5 | 3 | 1.13 |
| Cook Islands | . | 5 | 3 | 4 | . |

## **Appendix Table 2-C** Overall and component cigarette tax scores, 2014

| Country | 2014 | | | | |
|---|---|---|---|---|---|
| | **Absolute Price** | **Affordability Change** | **Tax Share** | **Tax Structure** | **OVERALL** |
| Costa Rica | 1 | 4 | 2.5 | 4 | 2.88 |
| Côte d'Ivoire | 1 | 4 | 0 | 1 | 1.50 |
| Croatia | 2 | 0* | 4 | 3 | 2.25 |
| Cuba | . | 1 | 4.5 | . | . |
| Cyprus | 2 | 3 | 4.5 | 3 | 3.13 |
| Czechia | 2 | 0* | 4.5 | 4 | 2.63 |
| Democratic People's Republic of Korea | . | . | 0 | 0 | . |
| Democratic Republic of the Congo | 0 | 0* | 1 | 1 | 0.50 |
| Denmark | 2 | 3 | 3.5 | 4 | 3.13 |
| Djibouti | 1 | 0* | 0 | . | . |
| Dominica | 1 | 0 | 0 | 4 | 1.25 |
| Dominican Republic | 3 | 0* | 2.5 | 4 | 2.38 |
| Ecuador | 2 | 3 | 3.5 | 5 | 3.38 |
| Egypt | 1 | 4 | 4.5 | 1 | 2.63 |
| El Salvador | 1 | 0 | 2 | 4 | 1.75 |
| Equatorial Guinea | 0 | 0 | 0 | 2 | 0.50 |
| Eritrea | . | 0 | 2.5 | 2 | . |
| Estonia | 3 | 4 | 4.5 | 3 | 3.63 |
| Eswatini | 4 | 0* | 1.5 | . | . |
| Ethiopia | 0 | 0 | 0 | 2 | 0.50 |
| Fiji | 3 | 0 | 1 | 1 | 1.25 |
| Finland | 2 | 3 | 4.5 | 3 | 3.13 |
| France | 3 | 3 | 4.5 | 3 | 3.38 |
| Gabon | 1 | 0 | 0 | 2 | 0.75 |
| Gambia | 1 | 5 | 1.5 | 4 | 2.88 |
| Georgia | 1 | 0* | 1.5 | 1 | 0.88 |
| Germany | 3 | 0 | 3.5 | 4 | 2.63 |
| Ghana | 1 | 0* | 0 | 2 | 0.75 |
| Greece | 2 | 5 | 4.5 | 4 | 3.88 |
| Grenada | 1 | 0 | 1.5 | 2 | 1.13 |
| Guatemala | 2 | 0 | 1.5 | 2 | 1.38 |
| Guinea | . | 0 | . | . | . |
| Guinea-Bissau | 0 | 0* | 0 | . | . |
| Guyana | 1 | 0* | 0 | 2 | 0.75 |
| Haiti | . | . | . | . | . |
| Honduras | 1 | 0 | 0.5 | 5 | 1.63 |
| Hungary | 3 | 4 | 4 | 3 | 3.50 |
| Iceland | 3 | 4 | 2 | 4 | 3.25 |

| | 2014 | | | | |
|---|---|---|---|---|---|
| Country | Absolute Price | Affordability Change | Tax Share | Tax Structure | OVERALL |
| India | 4 | 0 | 0.5 | 1 | 1.38 |
| Indonesia | 2 | 0* | 2 | 1 | 1.25 |
| Iran (Islamic Republic of) | 1 | 0 | 0 | 0 | 0.25 |
| Iraq | 0 | 0* | 0 | 0 | 0.00 |
| Ireland | 5 | 0 | 4 | 4 | 3.25 |
| Israel | 3 | 3 | 4.5 | 3 | 3.38 |
| Italy | 2 | 4 | 4 | 3 | 3.25 |
| Jamaica | 5 | 1 | 0.5 | 4 | 2.63 |
| Japan | 2 | 4 | 3 | 1 | 2.50 |
| Jordan | 1 | 3 | 4.5 | 1 | 2.38 |
| Kazakhstan | 1 | 0 | 0.5 | 4 | 1.38 |
| Kenya | 0 | 0* | 1.5 | 2 | 0.88 |
| Kiribati | 2 | 0* | 2 | 4 | 2.00 |
| Kuwait | 2 | 1 | 0 | 0 | 0.75 |
| Kyrgyzstan | 0 | 0 | 0.5 | 1 | 0.38 |
| Lao People's Democratic Republic | 1 | 0 | 0 | 1 | 0.50 |
| Latvia | 2 | 0 | 4 | 4 | 2.50 |
| Lebanon | 1 | 0 | 1 | 2 | 1.00 |
| Lesotho | 3 | 0 | 1.5 | 5 | 2.38 |
| Liberia | 0 | 0* | 0 | . | . |
| Libya | 2 | 1 | 0 | 0 | 0.75 |
| Lithuania | 2 | 0 | 4 | 4 | 2.50 |
| Luxembourg | 2 | 4 | 3.5 | 3 | 3.13 |
| Madagascar | 1 | 4 | 4.5 | 2 | 2.88 |
| Malawi | 3 | 5 | 0 | 4 | 3.00 |
| Malaysia | 4 | 0 | 3 | 4 | 2.75 |
| Maldives | 1 | 0 | 2 | 0 | 0.75 |
| Mali | 1 | 0* | 0 | 1 | 0.50 |
| Malta | 3 | 0 | 3.5 | 4 | 2.63 |
| Marshall Islands | 0 | 0 | 1.5 | 0 | 0.38 |
| Mauritania | 2 | 0 | 0 | 2 | 1.00 |
| Mauritius | 3 | 4 | 3.5 | 4 | 3.63 |
| Mexico | 2 | 3 | 3.5 | 3 | 2.88 |
| Micronesia (Federated States of) | 0 | 0 | 1.5 | 0 | 0.38 |
| Monaco | . | . | . | . | . |
| Mongolia | 1 | 0 | 3.5 | 4 | 2.13 |
| Montenegro | 1 | 5 | 4.5 | 3 | 3.38 |
| Morocco | 2 | 0 | 3.5 | 1 | 1.63 |
| Mozambique | 0 | 3 | 0 | 1 | 1.00 |

## Appendix Table 2-C Overall and component cigarette tax scores, 2014

| Country | 2014 | | | | |
|---|---|---|---|---|---|
| | Absolute Price | Affordability Change | Tax Share | Tax Structure | OVERALL |
| Myanmar | 1 | 0* | 2.5 | 2 | 1.38 |
| Namibia | 3 | 0 | 0.5 | 4 | 1.88 |
| Nauru | . | 0 | . | . | . |
| Nepal | 2 | 0 | 0 | 1 | 0.75 |
| Netherlands | 3 | 3 | 3.5 | 5 | 3.63 |
| New Zealand | 5 | 4 | 4.5 | 5 | 4.63 |
| Nicaragua | 1 | 0 | 0 | . | . |
| Niger | 1 | 0 | 0 | 2 | 0.75 |
| Nigeria | 1 | 0* | 0 | 2 | 0.75 |
| Niue | . | . | 2.5 | 0 | . |
| North Macedonia | 1 | 0* | 3.5 | 4 | 2.13 |
| Norway | 5 | 3 | 3 | 4 | 3.75 |
| Oman | 3 | 1 | 0 | 0 | 1.00 |
| Pakistan | 0 | 0 | 2.5 | 1 | 0.88 |
| Palau | 2 | 0 | 4 | . | . |
| Panama | 2 | 0 | 2.5 | 2 | 1.63 |
| Papua New Guinea | 3 | 0 | 0.5 | 1 | 1.13 |
| Paraguay | 0 | 0 | 0 | . | . |
| Peru | 1 | 0 | 0.5 | 4 | 1.38 |
| Philippines | 0 | 0 | 4 | 1 | 1.25 |
| Poland | 3 | 5 | 4.5 | 3 | 3.88 |
| Portugal | 3 | 4 | 3.5 | 4 | 3.63 |
| Qatar | 2 | 1 | 0 | 0 | 0.75 |
| Republic of Korea | 1 | 0* | 3 | 4 | 2.00 |
| Republic of Moldova | 1 | 4 | 1.5 | 1 | 1.88 |
| Romania | 3 | 5 | 4 | 4 | 4.00 |
| Russian Federation | 1 | 4 | 1.5 | 4 | 2.63 |
| Rwanda | 1 | 0* | 0 | 2 | 0.75 |
| Saint Kitts and Nevis | 2 | 0 | 0 | 2 | 1.00 |
| Saint Lucia | 1 | 0 | 2.5 | 4 | 1.88 |
| Saint Vincent and the Grenadines | 1 | 2 | 0 | 4 | 1.75 |
| Samoa | 2 | 2 | 2.5 | 4 | 2.63 |
| San Marino | 3 | 1 | 4.5 | . | . |
| Sao Tome and Principe | 0 | 0* | 0 | 2 | 0.50 |
| Saudi Arabia | 3 | 1 | 0 | 0 | 1.00 |
| Senegal | 1 | 0 | 0.5 | 1 | 0.63 |
| Serbia | 2 | 5 | 4.5 | 3 | 3.63 |
| Seychelles | 4 | 0* | 4.5 | 4 | 3.13 |
| Sierra Leone | 0 | 0 | 0 | 0 | 0.00 |
| Singapore | 5 | 0 | 3.5 | 4 | 3.13 |

| | 2014 | | | | |
|---|---|---|---|---|---|
| Country | Absolute Price | Affordability Change | Tax Share | Tax Structure | OVERALL |
| Slovakia | 2 | 3 | 4.5 | 4 | 3.38 |
| Slovenia | 2 | 5 | 4.5 | 4 | 3.88 |
| Solomon Islands | 1 | 0 | 0 | 1 | 0.50 |
| Somalia | . | . | . | . | . |
| South Africa | 2 | 0 | 1.5 | 5 | 2.13 |
| South Sudan | . | . | . | . | . |
| Spain | 3 | 5 | 4.5 | 3 | 3.88 |
| Sri Lanka | 5 | 0 | 3.5 | 1 | 2.38 |
| Sudan | 2 | 0 | 4 | 2 | 2.00 |
| Suriname | 2 | 2 | 2.5 | 4 | 2.63 |
| Sweden | 3 | 2 | 3 | 5 | 3.25 |
| Switzerland | 2 | 3 | 3 | 4 | 3.00 |
| Syrian Arab Republic | . | 0 | . | . | . |
| Tajikistan | 1 | 0 | 0 | 1 | 0.50 |
| Thailand | 2 | 0 | 4 | 2 | 2.00 |
| Timor-Leste | 0 | 0 | 0.5 | 4 | 1.13 |
| Togo | 0 | 0* | 0 | 2 | 0.50 |
| Tonga | 2 | 0 | 3 | 1 | 1.50 |
| Trinidad and Tobago | 2 | 5 | 0 | 4 | 2.75 |
| Tunisia | 1 | 1 | 3 | 3 | 2.00 |
| Turkey | 3 | 4 | 4.5 | 3 | 3.63 |
| Turkmenistan | 4 | 1 | 0 | 4 | 2.25 |
| Tuvalu | 0 | 0 | 0 | 2 | 0.50 |
| Uganda | 1 | 0 | 0.5 | 1 | 0.63 |
| Ukraine | 0 | 0 | 4.5 | 1 | 1.38 |
| United Arab Emirates | 2 | 1 | 0 | 0 | 0.75 |
| United Kingdom of Great Britain and Northern Ireland | 4 | 3 | 4.5 | 4 | 3.88 |
| United Republic of Tanzania | 2 | 0 | 0 | 1 | 0.75 |
| United States of America | 2 | 0 | 1 | 4 | 1.75 |
| Uruguay | 2 | 0 | 3 | 4 | 2.25 |
| Uzbekistan | 1 | 0 | 0 | 1 | 0.50 |
| Vanuatu | 2 | 0 | 2 | . | . |
| Venezuela (Bolivarian Republic of) | . | 1 | 4 | . | . |
| Viet Nam | 1 | 0* | 0 | 2 | 0.75 |
| West Bank and Gaza Strip | . | 5 | 4.5 | 4 | . |
| Yemen | . | 4 | 1 | 4 | . |
| Zambia | 1 | 0* | 0 | 2 | 0.75 |
| Zimbabwe | 1 | 0 | 0.5 | 4 | 1.38 |
| Global Average | 1.73 | 1.25 | 1.91 | 2.48 | 1.85 |

Note: Overall scores are rounded to two decimal places. Countries in each column are listed in order of their scores, from lowest to highest, and alphabetically when scores are identical.

## **Appendix Table 3** Overall cigarette tax scores, 2014, 2016, and 2018

| Country | Overall score (2014) | Overall score (2016) | Overall score (2018) |
|---|---|---|---|
| Afghanistan | 0.00 | 0.25 | 0.25 |
| Albania | 2.88 | 3.00 | 2.25 |
| Algeria | 0.88 | 2.38 | 2.50 |
| Andorra | . | . | . |
| Angola | 0.50 | . | 0.50 |
| Antigua and Barbuda | 0.75 | 0.50 | 0.50 |
| Argentina | 1.75 | 2.00 | 3.50 |
| Armenia | 0.50 | 0.63 | 0.88 |
| Australia | 4.13 | 4.25 | 4.63 |
| Austria | 2.88 | 2.75 | 2.50 |
| Azerbaijan | 1.00 | 3.00 | 0.63 |
| Bahamas | 3.38 | 3.50 | . |
| Bahrain | 0.75 | 1.00 | 3.75 |
| Bangladesh | 0.88 | 1.13 | 2.38 |
| Barbados | 2.63 | 2.63 | 2.13 |
| Belarus | 0.63 | 0.63 | 1.88 |
| Belgium | 3.25 | 3.25 | 3.25 |
| Belize | 0.63 | 1.63 | 1.50 |
| Benin | 0.75 | 0.75 | 0.75 |
| Bhutan | . | . | . |
| Bolivia (Plurinational State of) | 0.88 | 0.88 | 0.63 |
| Bosnia and Herzegovina | 3.63 | 3.88 | 3.63 |
| Botswana | 2.38 | 2.13 | 2.38 |
| Brazil | 1.88 | 2.88 | 2.13 |
| Brunei Darussalam | . | . | . |
| Bulgaria | 2.88 | 2.63 | 3.13 |
| Burkina Faso | 0.50 | 0.50 | 1.63 |
| Burundi | 1.38 | 1.38 | 1.38 |
| Cabo Verde | 0.75 | 0.75 | 0.75 |
| Cambodia | 0.50 | 0.50 | 0.50 |
| Cameroon | . | 0.75 | 0.75 |
| Canada | 3.00 | 3.13 | 3.75 |
| Central African Republic | 0.75 | . | 0.88 |
| Chad | 0.75 | 2.00 | 2.25 |
| Chile | 2.38 | 3.50 | 3.63 |
| China | 0.88 | 1.00 | 1.25 |
| Colombia | 1.38 | 1.63 | 2.38 |
| Comoros | 1.13 | 0.88 | 1.13 |
| Congo | 1.13 | 2.38 | 1.13 |
| Cook Islands | . | . | . |
| Costa Rica | 2.88 | 2.13 | 2.13 |

| Country | Overall score (2014) | Overall score (2016) | Overall score (2018) |
|---|---|---|---|
| Côte d'Ivoire | 1.50 | 1.50 | 0.75 |
| Croatia | 2.25 | 2.50 | 2.50 |
| Cuba | . | . | . |
| Cyprus | 3.13 | 3.13 | 2.88 |
| Czechia | 2.63 | 2.63 | 2.75 |
| Democratic People's Republic of Korea | . | . | . |
| Democratic Republic of the Congo | 0.50 | 0.25 | 0.63 |
| Denmark | 3.13 | 2.88 | 2.38 |
| Djibouti | . | 0.50 | . |
| Dominica | 1.25 | 1.25 | 1.25 |
| Dominican Republic | 2.38 | 2.38 | 2.38 |
| Ecuador | 3.38 | 4.50 | 4.38 |
| Egypt | 2.63 | 2.63 | 2.75 |
| El Salvador | 1.75 | 1.75 | 1.88 |
| Equatorial Guinea | 0.50 | 0.50 | 1.00 |
| Eritrea | . | . | . |
| Estonia | 3.63 | 3.63 | 2.88 |
| Eswatini | . | 2.38 | 2.25 |
| Ethiopia | 0.50 | 0.50 | 0.50 |
| Fiji | 1.25 | 1.50 | 2.75 |
| Finland | 3.13 | 3.38 | 3.38 |
| France | 3.38 | 3.63 | 3.63 |
| Gabon | 0.75 | 0.75 | 1.00 |
| Gambia | 2.88 | 2.88 | 3.13 |
| Georgia | 0.88 | 1.38 | 1.38 |
| Germany | 2.63 | 2.63 | 2.88 |
| Ghana | 0.75 | 0.75 | 0.75 |
| Greece | 3.88 | 3.88 | 3.63 |
| Grenada | 1.13 | 1.38 | 1.13 |
| Guatemala | 1.38 | 1.38 | 1.38 |
| Guinea | . | 0.50 | . |
| Guinea-Bissau | . | 0.50 | 0.75 |
| Guyana | 0.75 | 0.75 | 1.25 |
| Haiti | . | . | . |
| Honduras | 1.63 | 1.50 | 1.75 |
| Hungary | 3.50 | 3.88 | 2.88 |
| Iceland | 3.25 | 2.50 | 2.50 |
| India | 1.38 | 2.38 | 1.88 |
| Indonesia | 1.25 | 1.38 | 1.63 |
| Iran (Islamic Republic of) | 0.25 | 1.25 | 0.50 |
| Iraq | 0.00 | 0.00 | 0.00 |

## Appendix Table 3 Overall cigarette tax scores, 2014, 2016, and 2018

| Country | Overall score (2014) | Overall score (2016) | Overall score (2018) |
|---|---|---|---|
| Ireland | 3.25 | 3.25 | 3.25 |
| Israel | 3.38 | 3.88 | 3.63 |
| Italy | 3.25 | 2.50 | 3.00 |
| Jamaica | 2.63 | 2.88 | 3.13 |
| Japan | 2.50 | 1.50 | 1.50 |
| Jordan | 2.38 | 2.38 | 3.38 |
| Kazakhstan | 1.38 | 2.63 | 3.00 |
| Kenya | 0.88 | 1.63 | 0.88 |
| Kiribati | 2.00 | 1.75 | 1.75 |
| Kuwait | 0.75 | 0.75 | 0.75 |
| Kyrgyzstan | 0.38 | 0.63 | 2.88 |
| Lao People's Democratic Republic | 0.50 | 1.25 | 0.50 |
| Latvia | 2.50 | 2.63 | 2.88 |
| Lebanon | 1.00 | 1.00 | 0.88 |
| Lesotho | 2.38 | . | 2.13 |
| Liberia | . | 0.50 | 0.50 |
| Libya | 0.75 | 0.50 | 0.25 |
| Lithuania | 2.50 | 2.75 | 3.38 |
| Luxembourg | 3.13 | 2.88 | 2.38 |
| Madagascar | 2.88 | 3.13 | 2.13 |
| Malawi | 3.00 | . | . |
| Malaysia | 2.75 | 2.75 | 2.88 |
| Maldives | 0.75 | 0.75 | 1.25 |
| Mali | 0.50 | 0.50 | 0.50 |
| Malta | 2.63 | 3.13 | 3.13 |
| Marshall Islands | 0.38 | 0.50 | 0.50 |
| Mauritania | 1.00 | 0.75 | 0.75 |
| Mauritius | 3.63 | 2.63 | 3.25 |
| Mexico | 2.88 | 2.13 | 2.13 |
| Micronesia (Federated States of) | 0.38 | 0.88 | 0.75 |
| Monaco | . | . | . |
| Mongolia | 2.13 | 1.75 | 1.63 |
| Montenegro | 3.38 | 3.38 | 3.88 |
| Morocco | 1.63 | 1.63 | 2.38 |
| Mozambique | 1.00 | 0.25 | 0.50 |
| Myanmar | 1.38 | 0.75 | 0.63 |
| Namibia | 1.88 | 2.00 | 2.00 |
| Nauru | . | 1.50 | 1.50 |
| Nepal | 0.75 | 0.75 | 1.75 |
| Netherlands | 3.63 | 3.63 | 3.13 |
| New Zealand | 4.63 | 4.75 | 4.63 |

| Country | Overall score (2014) | Overall score (2016) | Overall score (2018) |
|---|---|---|---|
| Nicaragua | . | 1.63 | 1.63 |
| Niger | 0.75 | 0.88 | 0.75 |
| Nigeria | 0.75 | 0.75 | 0.75 |
| Niue | . | . | . |
| North Macedonia | 2.13 | 2.38 | 2.63 |
| Norway | 3.75 | 3.75 | 3.63 |
| Oman | 1.00 | 1.00 | 1.00 |
| Pakistan | 0.88 | 2.25 | 0.88 |
| Palau | . | 2.63 | 3.88 |
| Panama | 1.63 | 1.88 | 1.88 |
| Papua New Guinea | 1.13 | 1.63 | 1.75 |
| Paraguay | . | 0.50 | 0.50 |
| Peru | 1.38 | 3.13 | 3.63 |
| Philippines | 1.25 | 2.50 | 3.75 |
| Poland | 3.88 | 3.75 | 2.75 |
| Portugal | 3.63 | 2.63 | 2.88 |
| Qatar | 0.75 | 0.75 | 0.88 |
| Republic of Korea | 2.00 | 2.50 | 2.50 |
| Republic of Moldova | 1.88 | 1.13 | 1.13 |
| Romania | 4.00 | 2.88 | 3.13 |
| Russian Federation | 2.63 | 3.13 | 3.38 |
| Rwanda | 0.75 | 1.63 | 1.63 |
| Saint Kitts and Nevis | 1.00 | 1.00 | 1.00 |
| Saint Lucia | 1.88 | 1.88 | 1.88 |
| Saint Vincent and the Grenadines | 1.75 | 1.25 | 1.50 |
| Samoa | 2.63 | 2.63 | 3.13 |
| San Marino | . | . | . |
| Sao Tome and Principe | 0.50 | 0.50 | 1.25 |
| Saudi Arabia | 1.00 | 1.00 | 3.75 |
| Senegal | 0.63 | 0.88 | 1.63 |
| Serbia | 3.63 | 3.63 | 3.63 |
| Seychelles | 3.13 | 3.13 | 3.13 |
| Sierra Leone | 0.00 | 0.00 | 1.50 |
| Singapore | 3.13 | 3.13 | 3.25 |
| Slovakia | 3.38 | 3.38 | 2.88 |
| Slovenia | 3.88 | 3.38 | 2.88 |
| Solomon Islands | 0.50 | 0.50 | 0.75 |
| Somalia | . | . | . |
| South Africa | 2.13 | 1.88 | 2.50 |
| South Sudan | . | . | . |
| Spain | 3.88 | 2.63 | 2.63 |

## Appendix Table 3 Overall cigarette tax scores, 2014, 2016, and 2018

| Country | Overall score (2014) | Overall score (2016) | Overall score (2018) |
|---|---|---|---|
| Sri Lanka | 2.38 | 2.13 | 3.38 |
| Sudan | 2.00 | 2.00 | 2.00 |
| Suriname | 2.63 | 2.75 | 3.38 |
| Sweden | 3.25 | 3.00 | 2.75 |
| Switzerland | 3.00 | 3.00 | 2.50 |
| Syrian Arab Republic | . | . | . |
| Tajikistan | 0.50 | 0.50 | 2.38 |
| Thailand | 2.00 | 2.25 | 1.75 |
| Timor-Leste | 1.13 | 1.25 | 1.75 |
| Togo | 0.50 | 0.75 | 0.75 |
| Tonga | 1.50 | 2.75 | 3.63 |
| Trinidad and Tobago | 2.75 | 3.00 | 3.25 |
| Tunisia | 2.00 | 1.75 | 1.75 |
| Turkey | 3.63 | 2.63 | 2.88 |
| Turkmenistan | 2.25 | 2.00 | 3.00 |
| Tuvalu | 0.50 | 1.25 | 1.00 |
| Uganda | 0.63 | 0.75 | 0.75 |
| Ukraine | 1.38 | 2.88 | 3.13 |
| United Arab Emirates | 0.75 | 0.75 | 3.50 |
| United Kingdom of Great Britain and Northern Ireland | 3.88 | 3.88 | 4.38 |
| United Republic of Tanzania | 0.75 | 0.75 | 0.75 |
| United States of America | 1.75 | 2.00 | 2.00 |
| Uruguay | 2.25 | 2.25 | 2.50 |
| Uzbekistan | 0.50 | 0.63 | 0.88 |
| Vanuatu | . | 2.00 | 2.13 |
| Venezuela (Bolivarian Republic of) | . | . | . |
| Viet Nam | 0.75 | 0.88 | 0.88 |
| West Bank and Gaza Strip | . | . | . |
| Yemen | . | . | . |
| Zambia | 0.75 | 1.13 | 1.13 |
| Zimbabwe | 1.38 | 2.63 | 2.63 |

*www.tobacconomics.org*
*@tobacconomics*
